# Neural Computing Advancements in Cardiac Imaging: A Review of Deep Learning Approaches for Heart Disease Diagnosis

**DOI:** 10.3390/jimaging12050180

**Published:** 2026-04-22

**Authors:** Tarek Berghout

**Affiliations:** Laboratory of Automation and Manufacturing Engineering, Department of Industrial Engineering, Batna 2 University, Batna 05000, Algeria; t.berghout@univ-batna2.dz

**Keywords:** cardiovascular healthcare, deep learning, heart disease, medical images, neural networks

## Abstract

Heart disease remains a leading cause of mortality worldwide, and timely and accurate diagnosis is crucial for improving patient outcomes. Medical imaging plays a pivotal role in this process, yet traditional diagnostic methods often suffer from limitations, including dependency on manual interpretation, susceptibility to observer variability, and inefficiency in handling large-scale data. Deep learning has emerged as an innovative technology in medical imaging, providing unparalleled advancements in feature extraction, segmentation, classification, and prediction tasks. Despite its proven potential, comprehensive reviews of deep learning methods specifically targeted at cardiac imaging remain scarce. This review paper seeks to bridge this gap by analyzing the state-of-the-art deep learning applications for heart disease diagnosis, covering the period from 2015 to 2025. Employing a well-structured methodology, this review categorizes and examines studies based on imaging modalities: Ultrasound (US), Magnetic Resonance Imaging (MRI), X-ray, Computed Tomography (CT), and Electrocardiography (ECG). For each modality, the analysis focuses on utilized datasets, processing techniques (e.g., extraction, segmentation and classification), and paradigms (e.g., transfer learning, federated learning, explainability, interpretability, and uncertainty quantification). Additionally, the types of heart disease addressed and prediction accuracy metrics are also scrutinized. These findings point toward future opportunities, including the study of data quality, optimization, transfer learning, uncertainty quantification and model explainability or interpretability. Furthermore, exploring advanced techniques such as recurrent expansion, transformers, and other architectures may unlock new pathways in cardiac imaging research. This review is a critical synthesis offering a roadmap for researchers and practitioners to advance the application of deep learning in heart disease diagnosis.

## 1. Introduction

**Figure 1:**
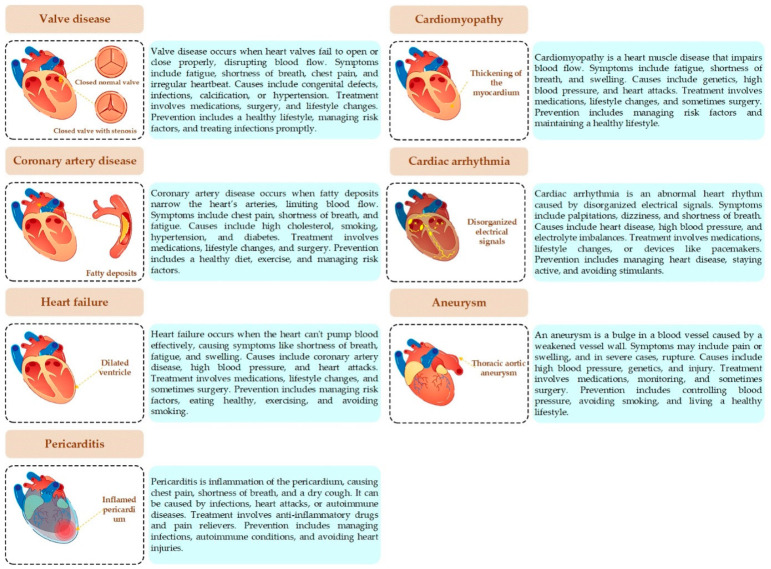
Descriptions of heart diseases, with an emphasis on symptoms, causes, treatment, and prevention methodologies. This figure is an original design prepared specifically for this paper. Only a few images are obtained from [6,7] which are under open-access licenses.

Heart disease is a leading cause of morbidity and mortality worldwide, affecting millions of people each year [1]. It encompasses a wide range of conditions that impact the heart’s structure and function, including coronary artery disease, heart failure, arrhythmias, valvular heart disease, congenital heart defects, cardiomyopathy, and pericardial disease (see Figure 1) [2]. Heart disease symptoms range from chest pain and fatigue to palpitations and dizziness, with causes including genetic, lifestyle, and metabolic factors [3]. Treatment varies from lifestyle modifications and medications to surgical interventions [3,4,5]. Prevention plays a critical role in reducing the risk of heart disease and involves managing risk factors such as maintaining a healthy weight, controlling blood pressure and cholesterol levels, staying physically active, and avoiding tobacco use. Regular check-ups and early detection can also help in preventing the progression of heart disease [2].

Accurate and early diagnosis is critical for effective treatment and management of heart diseases [3]. Medical imaging plays a vital role in diagnosing these conditions, allowing for detailed visualization of anatomy and function of the heart [8,9,10]. Imaging techniques such as ECG, MRI, X-rays, and CT are widely used to detect abnormalities, assess the severity of disease, and guide treatment decisions [11,12]. These imaging methods enable clinicians to observe the structure and functioning of the heart, identify blockages, assess the condition of heart valves, and monitor disease progression [11,12]. However, traditional image analysis methods, such as manual contouring, thresholding, edge detection, and region-growing techniques, while effective, have limitations. These methods often rely heavily on the experience of radiologists and technicians, which can lead to inconsistencies and errors, especially when analyzing complex or subtle abnormalities [13,14]. Furthermore, traditional image processing methods can be time-consuming and may not fully utilize the vast amount of data present in modern imaging techniques [13,14]. The emergence of deep learning has revolutionized the field of medical image analysis, offering a powerful alternative to traditional methods [15,16,17,18,19,20]. Deep learning algorithms, particularly Convolutional Neural Networks (CNNs), have demonstrated remarkable capabilities in automating the interpretation of medical images with high accuracy [15,16,17,18]. These models can learn from large datasets, recognize complex patterns, and make predictions without human intervention [21,22,23]. The transition to deep learning in heart disease diagnosis is increasingly necessary, as it can overcome the limitations of traditional image analysis methods, providing faster, more accurate, and consistent results [15,16,17,18,21,22,23]. More specifically in another way, accurate and timely diagnosis of heart disease remains a major global health priority. While traditional diagnostic tools like ECGs and clinical assessments are valuable, they may not capture the full structural and functional details of cardiac pathology. Cardiac imaging, such as echocardiography, MRI, and chest radiography, provides non-invasive, high-resolution insight into anatomical and physiological changes associated with cardiovascular disease. These modalities are especially critical for diagnosing conditions like heart failure, cardiomyopathies, and congenital defects. With the increasing availability of large image datasets and the advancement of deep learning, there is a strong opportunity to automate and enhance cardiac diagnosis by leveraging imaging data. This review is motivated by the need to assess and summarize how deep learning models applied to cardiac images can improve diagnostic performance, reduce clinical workload, and support real-time, scalable decision-making in diverse healthcare settings.

Recent theoretical reviews have explored the application of machine learning in heart disease diagnosis, with many discussing the use of various sensors to collect data for analysis [24,25,26]. While such reviews provide a broad understanding of machine learning applications, few focus specifically on medical imaging and deep learning. This is a critical gap, as images combined with deep learning applications are often more valuable in diagnosing heart disease. Unlike traditional machine learning-based methods, which may offer limited understanding, image-based deep learning provides rich, high-dimensional information that can reveal complex details of the heart’s structure and function. Therefore, this study is dedicated specifically to the use of images in heart disease diagnosis, highlighting the importance of deep learning techniques in this area. Given the rarity of reviews focused solely on image analysis, this work aims to fill that gap and provide a comprehensive overview of how deep learning is renovating the way heart disease is diagnosed and treated.

For this purpose, the introduction is structured into four key subsections. The first subsection explores the research methodology, detailing how the papers were systematically collected to ensure a reliable and rigorous approach. The second subsection reviews research trends and highlights significant gaps in the literature, providing a comprehensive overview of the current state of the field. The third subsection presents the contributions of this work, while the fourth subsection outlines the structure of the paper.

### 1.1. Research Collection

In well-structured research, the process of data collection and cleaning is a crucial step in ensuring the reliability and quality of the obtained research items in building the results of this review. The procedure typically involves multiple stages, each focusing on a specific aspect of the dataset. The first step involves combining data from multiple sources. In this study, data are collected from three distinct databases, spanning a decade of research (2015–2025): Scopus, PubMed, and Crossref. This is performed using Publish or Perish software [27]. Web of Science could not be explored in this study due to its requirement for external registration, which involves restricted access and associated licensing constraints. Google Scholar was also avoided because it collects and sometimes diverges research into numerous unpublished materials, lacking crucial details such as digital object identifier and research item type, which are essential for later data processing in our case.

In the first stage, we used keywords like “deep learning,” “heart,” “images,” “medical,” and “diagnosis,” with the title preferably including “heart.” Despite this, many irrelevant results were obtained in the area of research, and some were completely unrelated to the intended topics. Accordingly, by developing a MATLAB (Version: 23.2.0.2515942 (R2023b) Update 7) script, the relevant details, including the title, year, type, and digital object identifier, are extracted from each collected file of the software in the form of “.csv” files. Afterward, data from these files is merged into a single table, ensuring consistency. Simultaneously, unwanted entries, such as editorial pieces or retracted articles, are filtered out. The types of papers are standardized by converting various synonyms into a uniform classification, which helps maintain data consistency.

The second step focuses on removing duplicate entries. In datasets of academic research papers, such as the collected ones, it is common to encounter multiple records of the same article, often due to slight differences in formatting or metadata. To address this, duplicates are identified and removed based on the DOI, ensuring that each paper appears only once. This reduces redundancy and helps improve the accuracy of subsequent analyses.

The third step refines the dataset by filtering based on relevant keywords. In this case, the focus is on papers related to cardiovascular research. Titles containing specific heart-related terms, such as “cardiac,” “myocardial,” and “hypertension,” are retained, while others are excluded. This process narrows down the dataset to papers directly relevant to the research topic, enhancing the focus of the analysis.

The fourth step introduces an additional filter, focusing on image-related research. By searching for terms like “MRI,” “ultrasound,” and “radiograph,” the dataset is further refined to include only papers that involve imaging techniques, which are integral to many medical studies, particularly in cardiology. This selective filtering ensures that the final dataset contains studies related to both the subject of interest and the research methodologies being considered.

The fifth and final filtering step targets deep learning-related research. As artificial intelligence plays an increasing role in medical research, especially in imaging and diagnostics, the dataset is refined to include only those papers that mention terms associated with deep learning techniques, such as “CNN,” “Long Short-Term Memory (LSTM),” “Generative Adversarial Networks (GAN),” “Autoencoders,” and others. This step ensures the dataset focuses on cutting-edge methodologies, aligning with current trends in research.

Throughout these steps, the data are tracked and recorded in a step-tracking mechanism, which helps monitor the progress of the data cleaning process. After all filters are applied, the final cleaned dataset, along with the tracking information, is saved for further analysis, ensuring the data are ready for the paper collection and downloading process. The review methodology of paper collections started on 10 December 2024, and the entire downloading process was completed by 17 December 2024.

**Figure 2:**
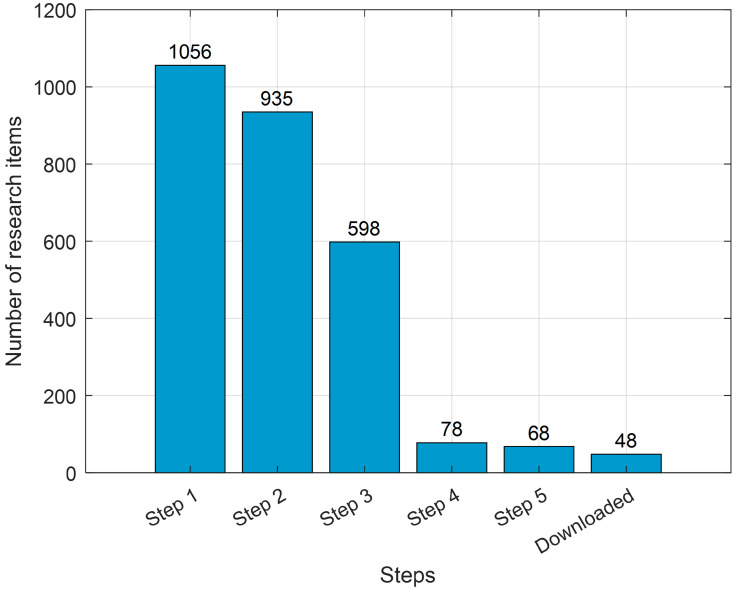
Research item counts across data cleaning and filtering steps.

Figure 2 illustrates the progressive refinement of research items through six data cleaning and filtering steps, showing the impact of each stage on dataset size. Starting with 1056 items after type cleaning, the count decreases to 935 after duplicate removal, 598 after filtering for heart-related titles, 78 for image-related studies, and 68 for deep learning-related titles. The final step, representing successfully downloaded papers, concludes with 48 items. This step-by-step process highlights a focused approach to building a high-quality analysis.

### 1.2. Research Trends and Important Gaps

This subsection provides an overview of the current research trends in the field and highlights the critical gaps that remain unaddressed, paving the way for exploration and innovation.

#### 1.2.1. Research Trends

**Figure 3:**
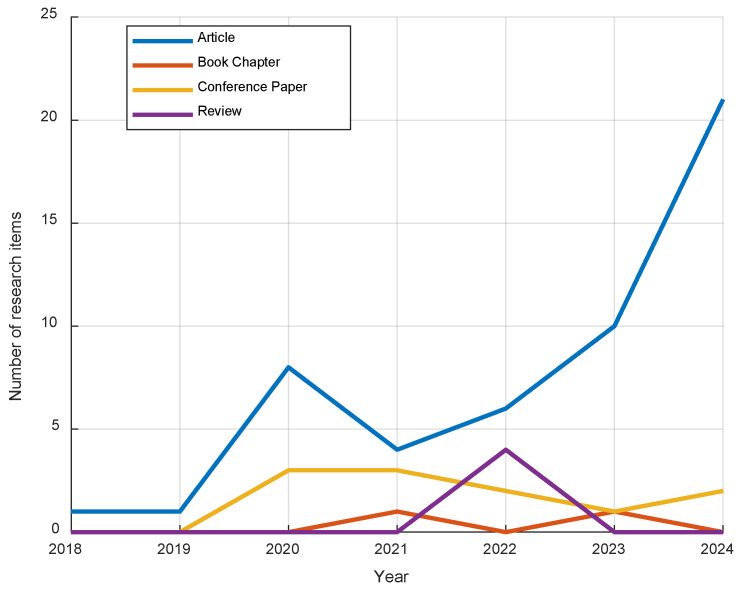
Trends in heart disease research with deep learning and medical imaging by type.

Figure 3 shows the research trends for various publication types from 2018 to 2024. Research articles demonstrate a significant upward trend, starting with only one item in 2018 and 2019, growing to eight in 2020, and reaching a peak of twenty-one in 2024. In contrast, book chapters show minimal activity, with items only recorded in 2021 and 2023, and none in 2022 and 2024. Conference papers follow a modest pattern, peaking at three items in 2020 and 2021 before declining in 2022 and 2023, with a slight increase in 2024. Reviews saw no activity until 2022, when they peaked at four items, but no further research items were recorded in 2023 or 2024. Overall, articles exhibit the most consistent growth, while other types of publications display more fluctuating and less frequent trends.

**Figure 4:**
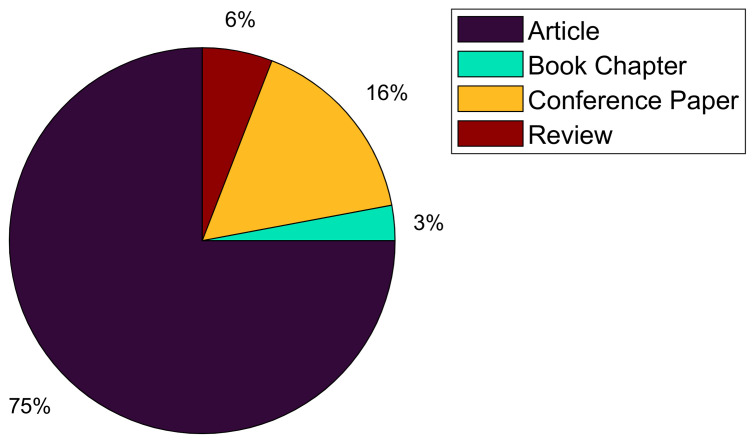
Distribution of heart disease research with deep learning and medical imaging by type.

Figure 4 depicts the distribution of research items by type, as shown in the pie chart: Articles (51 items), conference papers (11 items), reviews (4 items), and book chapters (2 items). The pie chart illustrates that research articles constitute the majority of the research items, followed by conference papers, reviews, and book chapters, which are relatively fewer in number. This distribution provides a clear visualization of the types of publications in the dataset, helping to highlight the prominence of articles in comparison to other types.

Overall, Figure 3 and Figure 4 highlight the dominance of articles in heart disease research with deep learning and medical imaging. Figure 3 shows steady growth in articles from 2018 to 2024, while other publication types fluctuate. Figure 4 confirms that articles make up the majority of the research items, underscoring their prominence in the field. Additionally, based on the titles of the research publications, the trend in this field of heart disease diagnosis and monitoring using deep learning and medical imaging is focused on applying deep learning techniques to a wide range of cardiac imaging modalities, such as US, MRI, X-rays, ECGs, and CT images. These studies predominantly emphasize the development of predictive models and automated systems for diagnosing heart conditions like heart failure, arrhythmias, and coronary artery disease, as well as assessing risks and outcomes like mortality and disease progression. Furthermore, there is significant interest in multimodal approaches, combining different imaging and diagnostic techniques (e.g., ECGs with chest X-rays or US) to improve the accuracy and robustness of heart disease detection. The research spans both adult and pediatric populations, with an increasing focus on innovative methodologies, including deep learning for real-time monitoring and personalized risk stratification.

#### 1.2.2. Research Gaps

A collection of selected reviews has been analyzed to identify gaps in building a comprehensive theoretical framework. In ref. [26], the authors reviewed state-of-the-art deep learning applications in cardiovascular image analysis, covering data types like cardiac MRI, CT, and US, and highlighting the use of CNNs and GANs. The review highlights advantages, including performance comparable to experienced physicians and potential clinical integration, but notes challenges in generalizability across domains. However, the review overlooked recent advancements, such as transformers, projected LSTMs, attention mechanisms, and transfer learning, which holds promise for improving adaptability and accuracy. Additionally, it neglects critical factors like data quality, compression, and their impact on learning, key issues given modern data complexity and constraints. While valuable, the review is somewhat outdated in addressing contemporary techniques and challenges. Future reviews should incorporate emerging architectures, address data-related challenges, and focus on strategies for improving model generalizability to stay aligned with current trends in deep learning-driven healthcare. In ref. [25], the authors reviewed deep learning techniques for cardiac image segmentation across imaging modalities like MRI, CT, and ultrasound, focusing on methods such as CNNs, Fully connected Networks (FCNs), and UNet architectures. The review classifies techniques based on heart diseases and components, including ventricles, atria, myocardium, and coronary vessels, highlighting their precision and reproducibility through public datasets. However, it overlooks complementary tasks like classification and feature extraction, as well as recent advancements such as transformers, attention mechanisms, and hybrid models that enhance segmentation accuracy and generalizability. The review also lacks discussion on preprocessing, data augmentation, and multimodal integration, critical for addressing data variability. The review would benefit from including these modern techniques and broader perspectives to better address challenges in cardiac image analysis. In ref. [24], the authors examined the role of artificial intelligence in diagnosing and detecting heart failure, discussing data sources like echocardiography, MRI, and ECGs, and techniques such as decision trees, CNNs, and RNNs. The paper highlights AI’s benefits, including improved diagnostic accuracy and early detection, while noting challenges like integration into clinical workflows. Although it explores advancements like ensemble neural networks for predictive analytics, the review primarily focuses on applications, with limited attention to image-specific tasks such as feature extraction, segmentation, and classification. A deeper focus on advanced deep learning techniques in cardiac imaging would have enhanced its impact. In ref. [11], the authors discussed multimodality cardiac image computing, providing a global overview of the field. However, deep learning is only briefly mentioned in a general sense, without a specific focus on its applications. This highlights a gap in the review, as it lacks a detailed exploration of deep learning techniques in cardiac image analysis. Table 1 provides a concise overview of the key aspects of each review.

In addition to the limitations highlighted in Table 1, it is important to note that previous works often provide limited details about the methodology behind paper collection and processing. In contrast, this review offers detailed visual representations, such as pie charts, bar charts, and illustrations, to document each step of the data collection and cleaning process, accompanied by an analysis of the results at every stage. This gap in the literature is crucial to address when developing a comprehensive theoretical framework. Moreover, many studies neglect to discuss advanced techniques, such as attention mechanisms, transformers, GANs, and autoencoders, even from a conceptual perspective. Furthermore, the quality and presentation of images and data are often overlooked, which can significantly impact on the reliability of the results. Another key area that is underexplored is the integration of explainability and interpretability, which are essential in applications with high stakes, such as healthcare, where trustworthiness and transparency are paramount. These aspects should be prioritized in future research to enhance the robustness and practical applicability of deep learning models in clinical settings.

### 1.3. Contributions

In addition to the comprehensive paper collection methodology outlined in Section 1.1 and the systematic extraction of research trends and gaps described in Section 1.2, which are significant contributions in themselves, this review introduces novel insights that advance the field substantially. Our primary focus is on categorizing and analyzing the collected papers based on the types of images employed in heart disease research.

1. **Categorization of image types:** Based on the analysis in Table 2, six distinct image categories were identified and systematically reviewed. These categories include US, X-ray, MRI, CT, ECG, and others (i.e., digital photos, microscopy, thermal images, audio, etc.). Together, they encapsulate the predominant imaging modalities explored in the reviewed studies.
2. **Systematic analysis:** Each group of papers in this review has been analyzed in a comprehensive and consistent manner based on a defined set of criteria. Specifically, the analysis considers (i) the datasets employed, including their source, size, diversity, and whether they are publicly available or institution specific; (ii) the methods applied for data preprocessing, feature extraction, segmentation, and classification; (iii) the paradigms adopted, such as transfer learning, federated learning, reinforcement learning, multi-task learning, explainability, interpretability, and uncertainty quantification; (iv) the specific types of cardiac diseases targeted, such as congenital heart disease, valvular disease, ischemic heart disease, cardiomyopathy, and heart failure; and (v) the performance metrics reported, including accuracy, sensitivity, specificity, AUROC, Dice similarity coefficient, Intersection over Union, and Hausdorff Distance. By systematically applying these criteria, the review ensures that comparisons across studies account for differences in datasets, methodological approaches, and disease contexts, while also identifying research gaps and opportunities for future advancement.
3. **Results gathering and discussion:** For each image type, the findings will be organized into a detailed table, offering a structured overview of the results. This will be complemented by thorough discussions and critical analyses that provide deeper insights into the methods and outcomes of the reviewed works.
4. **Global insights and future directions:** Building on these findings, the review will highlight key research gaps and challenges, such as the need for more advanced techniques, better integration of diverse imaging modalities, and the application of real-explainability and interpretability in healthcare models, in addition to tackling privacy-preserving issues and image compression impacts. By addressing these challenges, the review aims to pave the way for future innovations and impactful research directions.

This structured approach aims to bridge existing gaps in the literature and draws a clear path for future innovations in heart disease research. Emphasizing advanced methodologies and fostering the integration of diverse imaging modalities will aim to inspire robust, interpretable, and impactful solutions for healthcare challenges.

### 1.4. The Structure of the Review

**Figure 5:**
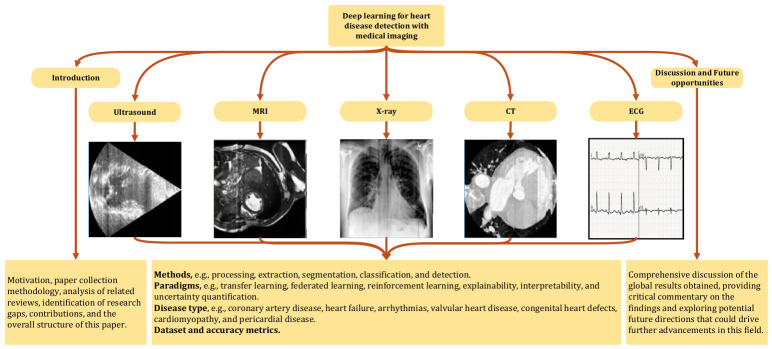
Diagram illustrating the structure and various sectors of this review study.

To provide a clearer understanding of the current state of deep learning and medical imaging, this review is structured into nine main sections. Beyond the introduction in Section 1, which covers the motivation, paper collection methodology, analysis of related reviews, identification of research gaps, contributions, and the overall structure of this paper, Section 2, Section 3, Section 4, Section 5, Section 6 and Section 7 systematically analyze existing methods. These sections are organized based on the frequency of usage of different imaging modalities in research papers, starting with US, followed by MRI, X-ray, CT, ECG, and other less commonly used image types. Section 8 presents a comprehensive discussion of the global results obtained, providing critical commentary on the findings and exploring potential future directions that could drive further advancements in this field. Finally, the review concludes in Section 9, summarizing key insights and implications. Figure 5 introduces an overview of the structure and various sections of this review study, providing a clear framework for understanding its organization and focus areas.

It should be emphasized that the objective of the present review is not to present an exhaustive architectural analysis of deep learning models, but rather to examine their use and relevance in cardiac imaging applications. Accordingly, the neural network architecture addressed in this manuscript are treated as established methods that are generally familiar to readers in this field. Readers seeking a broader introductory overview of commonly used architectures may refer to the survey by [69] which provides a comprehensive discussion of major supervised learning architectures used in medical image processing.

## 2. Trends in Ultrasound Imaging

This section introduces three main subsections: a brief overview and examples of US and deep learning, related works analysis, and research gaps and challenges. It begins with an overall definition of US imaging, emphasizing its significance in medical diagnostics, particularly in cardiac care. The section discusses the challenges faced in US imaging, such as image quality variability and noise. Illustrative examples are provided through realistic datasets, showcasing how deep learning techniques improve and tackle US image interpretation, often revealing details not visible to the naked eye. Furthermore, the section delves into an analysis of related works, exploring existing studies in ultrasound imaging and deep learning, identifying potential gaps, and highlighting emerging trends in the field.

### 2.1. Overview of Ultrasound Imaging and Deep Learning

In cardiology, echocardiography is essential for assessing heart structure, function, and blood flow, particularly for detecting coronary artery disease, valve abnormalities, cardiomyopathies, and congenital defects [70,71].

**Figure 6:**
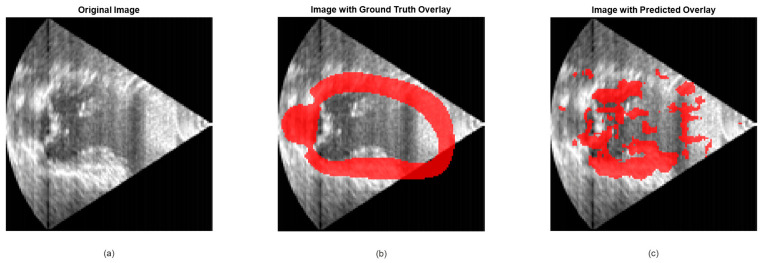
Example of echocardiographic image and deep learning-based segmentation: (**a**) Original echocardiographic image in the 2CH view at ED; (**b**) original image with overlaid segmentation mask highlighting the left ventricle; (**c**) predicted segmentation mask generated by the deep learning model, demonstrating close alignment with the ground truth.

Figure 6 demonstrates the utility of US and the potential application of deep learning in cardiac diagnostics. For this illustrative example, we selected images from one patient of the Cardiac Acquisitions for Multi-structure Ultrasound Segmentation (CAMUS) dataset, which includes echocardiographic images and corresponding ground truth annotations for the left ventricle and myocardium in the two-chamber (2CH) and four-chamber (4CH) views at end-diastole (ED) and end-systole (ES) phases [72].

The goal of this dataset was to provide all the materials to the community to address the challenge of echocardiographic image segmentation and volume estimation from 2D US sequences (both 2CH and 4CH views). In this context, the largest publicly available and fully annotated dataset for 2D echocardiographic assessment (according to the developers’ knowledge) [72]. The CAMUS dataset contains 2D apical 4CH and 2CH view sequences acquired from 500 patients and is made available to support research in this field.

For this study, we have only used a few examples, specifically, 50 2CH images from a single patient, for illustrative purposes. These images and masks were resized to a target size of 128 × 128 pixels for consistency and computational efficiency, and normalized to the range [0, 1] to ensure uniformity in intensity values, which is critical for training deep learning models. The designed model is a CNN tailored for cardiac image segmentation. It consists of convolutional layers with ReLU activations, max-pooling layers for downsampling, and transposed convolutional layers for upsampling. The model uses a sigmoid activation in the final layer to produce a binary mask and is trained using a mean squared error loss function. Bayesian optimization is employed to tune hyperparameters such as learning rate, number of filters, and mini-batch size for optimal performance [23].

Figure 6a displays an echocardiographic image in the 2CH view at ED, highlighting key anatomical structures such as the left ventricle and myocardium. The grayscale intensity represents US reflections, with brighter regions indicating stronger reflections (e.g., from the myocardium or valves). This visualization provides a clear representation of the raw imaging data used for analysis. Figure 6b overlays the ground truth segmentation mask on the original image, using a red transparent overlay to delineate the region of interest (e.g., the left ventricle). The ground truth masks were manually annotated by clinical experts and serve as the gold standard for evaluating the performance of automated segmentation algorithms. The red overlay highlights the accuracy of these annotations, providing a visual reference for the anatomical structures of interest.

Figure 6c showcases an example of deep learning-based prediction of the segmentation mask. The predicted mask is overlaid in red, demonstrating both the capacity of the model and the challenges in achieving complete coverage that matches the ground truth labels. While the model shows promising alignment with the ground truth in many regions, there are areas where the predicted mask fails to fully encompass the anatomical structures, particularly in regions with low contrast or ambiguous boundaries. This closeness highlights a set of challenges inherent in automated segmentation tasks, including variability in image quality, the complexity of cardiac structures, and the limitations of the model in handling edge cases. These challenges underscore the need for further refinement of the model, potentially through advanced architecture, larger and more diverse datasets, and post-processing techniques to improve segmentation accuracy and robustness.

Critically, such segmentation errors have direct clinical consequences. Inaccurate delineation of the left ventricle, as shown in Figure 6c, can lead to incorrect estimation of ventricular volumes and ejection fraction, which are key metrics for diagnosing heart failure, guiding therapy decisions, and predicting patient outcomes. For instance, underestimation of end-systolic volume may mask reduced systolic function, delaying necessary interventions, while overestimation could lead to unnecessary treatments or procedures. These clinical stakes underscore why improving segmentation accuracy is not merely a technical exercise but a prerequisite for safe and effective deployment of deep learning in cardiology.

### 2.2. Related Works of Ultrasound Imaging and Deep Learning

Recent state-of-the-art works have demonstrated the potential of deep learning to revolutionize cardiac ultrasound imaging.

For instance, the authors in [28] employed a dataset comprising 25,776 patients from two hospitals, with 1026 in-hospital mortalities, derived from echocardiography reports. The dataset was split into derivation data from Hospital A (20,651 patients) and external validation data from Hospital B (1560 patients). The methods applied included text mining to extract 11 continuous and 54 categorical predictor variables from echocardiography reports, followed by the development of a deep learning model using TensorFlow with three hidden neural network layers, batch normalization, and dropout layers. The study compared the deep learning model with logistic regression and random forest models. Specific paradigms such as transfer learning, federated learning, reinforcement learning, or explainability were not mentioned, though the authors acknowledged the black box nature of deep learning and expressed interest in future research on interpretable deep learning. The study addressed various heart diseases, including Coronary Heart Disease (CHD) and Heart Failure (HF), as indicated by the 10th revision of the International Classification of Diseases codes [73]. The prediction accuracy metrics reported were the Area Under the Receiver Operating Characteristic Curve (AUROC) and the Area Under the Precision-Recall Curve (AUPRC), with the deep learning model achieving AUROC values of 0.912 for internal validation, 0.898 for external validation, 0.958 for CHD, and 0.913 for HF, significantly outperforming other models.

The authors in [29] utilized a dataset comprising 1149 fetal heart images collected from ultrasound videos of pregnant women between 18 and 24 weeks of gestation. The dataset included four standard fetal heart views: 4CH, Three-Vessel and Trachea View (3VT), Left Ventricular Outflow Tract View (LVOT), and Right Ventricular Outflow Tract View (RVOT). It also investigates three types of CHDs: Atrial Septal Defect (ASD), Ventricular Septal Defect (VSD), and Atrioventricular Septal Defect (AVSD). The methods applied involved an instance segmentation approach using Mask Region-based CNN (R-CNN) with Residual Network with 50 layers (ResNet50) as the backbone for feature extraction, classification, and segmentation. The process included data acquisition, image annotation using LabelMe [74], and multi-task learning for simultaneous segmentation, classification, and detection of fetal heart structures and defects. The study employed transfer learning by pre-training the model on the Microsoft COCO dataset before fine-tuning it on the fetal heart dataset. As mentioned, the primary heart diseases addressed were congenital heart defects, ASD, VSD, and AVSD. The accuracy metrics reported included a Mean Average Precision (MAP) of 98.30% for intra-patient variation and 82.42% for inter-patient variation, with an Intersection Over Union (IOU) of 79.97% and a Dice Similarity Coefficient (DSC) of 89.70% for standard view segmentation. These results demonstrated the model’s effectiveness in accurately segmenting and detecting fetal heart structures and defects.

In the study carried out by the authors of [35], a dataset comprising 259 elderly patients diagnosed with sarcopenia, who underwent cardiac ultrasound examinations between October 2017 and March 2020, is utilized. The dataset was divided into two groups: a control group with unprocessed images and an experimental group with images processed using a CNN algorithm. The methods applied involved the use of a CNN model to optimize and enhance color Doppler US images, focusing on feature extraction and image reconstruction through convolutional layers, activation functions (ReLU and Swish), and loss functions for error correction. The study employed deep learning for image processing but did not explicitly mention paradigms such as transfer learning, federated learning, reinforcement learning, or explainability. The primary heart disease addressed was Chronic Heart Failure (CHF), with a focus on its correlation with sarcopenia. Accuracy metrics included the Peak Signal-to-Noise Ratio (PSNR = 20) and Structural Similarity Index Measure (SSIM = 0.09) for image quality, and the similarity between the final diagnosis and experimental group results was 93.5%, significantly higher than the control group (87.0%).

The contribution presented in [36] utilizes a dataset consisting of 161 Transthoracic 3D Echocardiograms (TTE 3DE) from 129 unique patients with Hypoplastic Left Heart Syndrome (HLHS), a severe congenital heart defect. The dataset was divided into 133 images for training and 28 for testing, with images acquired using Philips IE33 and EPIQ 7 ultrasound systems. The methods applied include FCN-based V-Net architecture for segmenting tricuspid valve (TV) leaflets. The FCN was trained using various input configurations, including single-phase, two-phase, four-phase, and Consecutive Systolic Phases (CSP), with additional inputs such as annular curves and commissural landmarks. The study employed data preprocessing techniques like isotropic resampling and standard orientation and used evaluation metrics such as DSC and Mean Boundary Distance (MBD) to assess segmentation accuracy. The paper does not explicitly mention the use of paradigms like transfer learning, federated learning, reinforcement learning, explainability, interpretability, or uncertainty quantification. The primary heart disease addressed is HLHS, with a focus on tricuspid valve segmentation. The reported accuracy metrics include a median DSC of 0.86 and MBD of 0.35 mm for merged segmentation, and an average DSC of 0.77 and MBD of 0.6 mm for individual TV leaflet segmentation. The addition of commissural landmarks improved individual leaflet segmentation accuracy to an MBD of 0.38 mm.

In ref. [37], the developed work adopts a dataset consisting of 80 elderly patients with Acute Left Heart Failure (ALHF) admitted to Affiliated Hangzhou First People’s Hospital from August 2017 to February 2019. The methods applied include a CNN algorithm for image preprocessing, which involves binarized threshold segmentation for denoising and illumination processing to balance image brightness. The CNN model is used for feature extraction and classification, with layers including convolution, activation functions, pooling, and fully connected layers. The primary heart disease addressed is ALHF. The reported accuracy metrics include a diagnostic coincidence rate of 93.94% for the observation group using deep learning-based echocardiography, compared to 74.29% for the control group using routine echocardiography. Additionally, the specificity, sensitivity, and accuracy of the deep learning-based method were higher than those of the control group, with statistically significant differences (*p* < 0.05).

In the study mentioned in [38], the authors utilize three datasets: the EchoNet-Dynamic dataset (10,030 echocardiographic videos), CAMUS dataset (450 ECG videos), and a local dataset from the National Cardiovascular Center of China. The methods applied include a deep spatio-temporal convolutional model, which is trained on dynamic data and applied to static data using regression training combined with a classification application approach. The model processes dynamic and static cardiac US images to identify HF with reduced Ejection Fraction (EF < 40%). The heart disease addressed is Heart Failure with reduced Ejection Fraction (HFrEF). The reported accuracy metrics include an AUROC of 0.95 on the dynamic EchoNet-Dynamic dataset and an AUROC of 1 on the independent validation set. For static data, the classification accuracies were 85%, 81%, 93%, and 92% when one, two, four, and eight images of the same heart were input, respectively. The model’s performance was comparable to the best-performing ultrasonographers and cardiologists with over 3 years of experience (*p* = 0.344) and significantly better than the median level (*p* = 0.000008).

The authors in [39] utilized the CAMUS dataset, with echocardiographic images from 450 patients, including apical 2CH and 4CH views at end-systole and end-diastole. The methods applied include a deep learning-based tool combining YOLOv7 algorithm for chamber identification and a UNet for segmentation. YOLOv7 algorithm detects the Left Atrium (LA) and Left Ventricle (LV), followed by image cropping and resizing, and the UNet performs segmentation of the Left Ventricular Endocardium (LVENDO), Epicardium (LVEPI), and LA. The study does not mention the use of paradigms federated learning, reinforcement learning, explainability, interpretability, or uncertainty quantification. The primary focus is on segmenting anatomical structures of the left heart, which is crucial for evaluating cardiac functionality, including conditions like HF and Myocardial Ischemia (MI). The reported accuracy metrics include DSC of 92.63% for LVENDO, 85.59% for LVEPI, and 87.57% for LA, along with Jaccard Index (JAC) and Hausdorff Distance (HD) values, demonstrating the tool’s reliability in segmenting left heart structures.

In ref. [40], the authors utilize a dataset consisting of 511 fetal heart Three-Vessel View (3VV) US images obtained from Hangzhou Normal University Affiliated Xiaoshan Hospital, China. The dataset includes 413 normal cases and 98 abnormal cases, with various types of CHD such as abnormal vessel diameter ratio, cardiac chamber abnormality, arterial vascular abnormality, outflow tract abnormality, and Tetralogy of Fallot. The methods applied involve a two-stage deep learning framework: the first stage uses Yolov5 for Region of Interest (ROI) localization, and the second stage employs a modified Deeplabv3 model equipped with an Attentional Multi-scale Feature Fusion (AMFF) module for fine segmentation of the three vessels (pulmonary artery, aorta, and superior vena cava). The accuracy metrics reported include DSC of 85.55%, 89.12%, and 77.54% for the pulmonary artery, aorta, and superior vena cava, respectively, along with an average Hausdorff Distance (HD) of 3.25 and an IOU value of 74.51%.

Reference [41] involves a dataset consisting of 153 2D US images of the heart LV collected from 18 patients (12 from the Institute of Cardiovascular Diseases, Vojvodina, and 6 from Newcastle University and Newcastle upon Tyne Hospitals NHS Foundation Trust). The dataset includes images from patients with cardiomyopathy, and data augmentation techniques such as mirroring were applied to increase the training set size. The methods applied involve the use of a UNet CNN for automatic segmentation of the LV. The UNet architecture consists of a contraction path for feature extraction and an expansion path for upsampling, with cross-over connections to preserve localization information. The network was trained using stochastic gradient descent with a learning rate of 0.002 and a regularization factor of 0.005. The primary heart disease addressed in the study is cardiomyopathy, specifically focusing on the asymmetrical pattern of LV hypertrophy. The accuracy metrics reported include a DSC of 83.49% for 128 × 128 images and 83.40% for 1016 × 708 images with kernel processing, along with HD and JC for evaluating segmentation performance.

The authors in [42] investigated a dataset consisting of 617,338 ECGs paired with Transthoracic Echocardiography (TTE) of 123,096 patients across five Mount Sinai hospitals in New York City. The dataset includes a demographically diverse cohort and focuses on detecting left heart valvular dysfunction, specifically Aortic Stenosis (AS) and Mitral Regurgitation (MR). The methods applied involve a natural language processing pipeline to extract ground-truth labels from TTE reports, followed by deep learning combining a multi-layer perceptron (MLP) and an EfficientNet for classification. The study employs group-stratified k-fold cross-validation to ensure robust model evaluation and uses saliency mapping for model interpretability, highlighting the importance of QRS complexes in predictions. The primary heart diseases addressed are moderate-to-severe AS and MR, with the DL model achieving an AUROC of 0.89 for AS and 0.88 for MR in internal testing, and 0.86 for AS and 0.81 for MR in external validation.

The authors in reference [30] conducted a study using a retrospective cohort from Taiwan (2011–2022) to differentiate three non-ischemic cardiomyopathies, Fabry Cardiomyopathy (FC), Hypertrophic Cardiomyopathy (HCM), and Cardiac Amyloidosis (CA), all presenting with Left Ventricular Hypertrophy (LVH). They utilized Transthoracic Echocardiography (TTE) as the imaging modality and employed a four-step automated deep learning approach, consisting of image quality assessment and view classification using a VGG16 model, auto-segmentation of the Interventricular Septum (IVS) based on UNet, and dynamic radiomics analysis using a LSTM model for classification. This workflow aimed to extract dynamic radiomic features from TTE images to identify the etiologies of hypertrophic phenocopies. The study emphasized deep learning paradigms such as interpretability through radiomics and segmentation techniques. The heart diseases addressed include FC, HCM, and CA. Accuracy metrics revealed 91.7% for image quality assessment, 95.2% for view classification, a DSC of 0.88 for auto-segmentation, and 74.5% accuracy for the LSTM model in classifying the three cardiomyopathies.

The authors in [31] utilized a dataset comprising echocardiographic acquisitions from 50 patients with sinus node dysfunction and implanted programmable pacemakers. The dataset included recordings of nine-loops with 10 cardiac cycles for the 4CH, 2CH, and apical long-axis views, acquired at baseline heart rates and during stepwise heart rates increments from 50 to 140 beats/min. The methods applied involved fully automated Left Ventricular Global Longitudinal Strain (LVGLS) measurements using a deep learning method developed by the authors. The deep learning method analyzed approximately 13,000 cardiac cycles, and a linear mixed model was used to construct an overall trendline, with patient number as the random effect. The study focused on automated deep learning for precise cardiac diagnostics. The primary heart condition addressed was Sinus Node Dysfunction (SND), with preserved atrioventricular conduction in all patients. The accuracy metrics highlighted a strong correlation between heart rates and LVGLS, with a 32% reduction in absolute LVGLS as heart rates increased from 50 to 140 beats/min, supported by a highly significant likelihood ratio test (*p*-value < 0.001).

The study developed in [32] discusses a dataset consisting of 91 routine clinical cardiac screening videos from 12 subjects, with gestational ages ranging from 20 to 35 weeks. The dataset included videos of the fetal heart, specifically focusing on three standard views: 4CH, LVOT, and 3V views. The methods applied involved CNNs for spatio-temporal feature aggregation, including Spatial Baseline Models (SBM) based on AlexNet and VGGNet, Direct Temporal Encoding (DTE), and Hierarchical Temporal Encoding (HTE). The HTE model progressively encoded temporal information throughout the network, enabling multi-task learning for joint prediction of visibility, view plane, and localization of the fetal heart. The study employed multi-task learning but did not explicitly mention federated learning, reinforcement learning, or explainability. The primary focus was on CHD detection through fetal heart analysis in ultrasound videos. Accuracy metrics included classification accuracy for view planes (e.g., 88.27% for LVOT and 79.80% for 3V using HTE) and localization accuracy with an IOU threshold of 0.25, achieving a localization error of 20.32% with HTE, significantly outperforming previous methods.

The work in [33] involves a dataset comprising 2556 echocardiography studies paired with Cardiac Magnetic Resonance (CMR) findings from 1453 patients, with studies conducted within 30 days of each other. The dataset included echocardiography views such as apical 4CH, apical 2CH, and Parasternal Long Axis (PLAX). The methods applied involved a video-based CNN with residual connections and spatio-temporal convolutions to predict CMR-derived labels, including Wall Motion Abnormalities (WMA), Late Gadolinium Enhancement (LGE), and abnormal T1, T2, and extracellular volume (ECV) values. The model was trained using binary cross-entropy loss for classification tasks and mean squared error for regression tasks, with early stopping and AdamW optimizer. The heart diseases addressed included conditions associated with myocardial fibrosis, inflammation, and infiltration, as identified by CMR. Accuracy metrics reported included an AUROC of 0.873 for predicting WMA, but lower performance for LGE (AUROC 0.699), T1 (AUROC 0.614), T2 (AUROC 0.553), and ECV (AUROC 0.564), indicating limited ability to predict CMR-based tissue characteristics from echocardiography.

In ref. [34], the authors utilize two datasets for their study: the first dataset consists of coronary CT angiography (CTA) images from 34 subjects without vascular centerline information, while the second dataset, provided by the Rotterdam Coronary Artery Algorithm Evaluation Framework, includes data from 18 subjects with centerline information. The methods applied involve an improved 3D UNet convolutional neural network for coronary artery segmentation, combined with a deep belief network (DBN) for feature extraction and regression of contour coordinates. The study employs data augmentation strategies and an overlap-tile strategy to enhance the training process. The primary heart disease addressed is Coronary Artery Disease (CAD), specifically focusing on the segmentation of coronary arteries to detect plaque and stenosis. The accuracy of the segmentation is evaluated using the Dice coefficient, with the best performance reaching a DSC of 0.8291.

Table 3 summarizes related works on US imaging and deep learning for heart disease detection. The current table structure facilitates better information extraction and improves clarity. It demonstrates the potential of deep learning in advancing cardiac imaging, particularly in US imaging, highlighting its adaptability, accuracy, and clinical relevance. Collectively, the studies in Table 3 demonstrate that deep learning achieves consistently high performance across diverse ultrasound-based tasks, though direct comparisons are limited by heterogeneous datasets and evaluation protocols.

### 2.3. Discussion of Research Gaps in Ultrasound Imaging and Deep Learning

Despite the significant advances in the field of cardiac image extraction, segmentation, and classification, reducing the accuracy gap (ranging from 0.81 to 0.95) remains a critical challenge. In this context, several gaps have been identified in the current methodologies. Firstly, many existing works do not explore advanced learning paradigms such as transfer learning, explainability, interpretability, and uncertainty quantification. While some studies indirectly introduce interpretability methodologies and a few employ transfer learning, often pre-training models on large datasets like Microsoft COCO to boost performance, these aspects are not systematically explored or leveraged to their full potential. Additionally, CNNs dominate the field, while other powerful feature extraction techniques like autoencoders and time-adaptive models for sequential data (e.g., age-related or temporal data) remain underutilized. Recent advancements in deep learning, such as GANs, transformers, and recurrent expansion algorithms [22,23,75], which could offer improved performance and flexibility, are also not sufficiently targeted. Furthermore, critical aspects of data privacy and distributed learning, such as federated learning, are largely overlooked, despite their potential to address privacy concerns in medical data. Similarly, reinforcement learning, which could optimize decision-making process tasks, is not explored. Addressing these gaps could lead to more robust, interpretable, and privacy-preserving models, ultimately improving the accuracy and applicability of cardiac imaging-based clinical practice.

When comparing ultrasound-based studies, several trends emerge that illustrate both the strengths and the limitations of current deep learning applications in this domain. Early large-scale works such as [28] demonstrated that deep learning applied to textual and imaging data from over 25,000 patients could achieve high AUROC values (up to 0.958 for CHD), emphasizing the feasibility of population-scale deployment. Fetal ultrasound-focused studies like [29] adopted transfer learning and multi-task learning approaches, achieving DSC values close to 90% and MAP scores exceeding 98%, thereby showing strong potential for early CHD detection in utero. More recent works such as [30] integrated multi-step pipelines combining CNNs, UNets, and LSTMs, providing interpretability through radiomics and temporal analysis, albeit with more moderate classification accuracies (around 74.5%). Echocardiographic segmentation studies, including [36], applied advanced architectures such as V-Net, YOLOv7, and UNet, reaching median DSC values between 0.83 and 0.93, underscoring the maturity of segmentation pipelines in echocardiography. At the same time, studies like [38,42] highlighted the use of multimodal data (e.g., TTE paired with ECGs, or echocardiography combined with CMR) and saliency-based interpretability, demonstrating AUROC values around 0.89 but also revealing performance variability across internal versus external validation. Collectively, the ultrasound studies reveal that deep learning can achieve high diagnostic performance across diverse use cases, from fetal CHD screening to adult heart failure and valvular disease, yet they also highlight the variability of results depending on dataset size, imaging quality, and validation strategy. The comparison suggests that while ultrasound imaging benefits strongly from CNN-based segmentation and transfer learning, future improvements may depend on more systematic adoption of advanced paradigms (e.g., transformers, GANs, federated learning) to boost generalizability, interpretability, and privacy-preserving applications.

While the reviewed ultrasound-based deep learning studies demonstrate strong diagnostic potential, latency and computational efficiency remain critical considerations for clinical adoption. For example, large-scale models such as those in [28] achieved high AUROC values but were primarily evaluated on centralized computational infrastructures, leaving open questions regarding their feasibility in real-time or bedside deployment. Similarly, advanced multi-step pipelines, such as those integrating CNNs, UNets, and LSTMs [30], provided interpretability and temporal analysis but at the cost of increased complexity and slower inference times, which may hinder use in urgent clinical settings. Echocardiographic segmentation frameworks like YOLOv7 and V-Net [36] achieved high DSC values but often required GPUs with substantial memory capacity, limiting their portability to resource-constrained environments. Moreover, multimodal pipelines combining ECG and echocardiographic data [42] improved performance but introduced additional synchronization and preprocessing overhead, which could contribute to latency during real-time analysis. These limitations indicate that, while accuracy is consistently strong across studies, further efforts are needed to streamline architectures, optimize inference speed, and validate deployment on low-power platforms to ensure that deep learning models can be reliably translated into point-of-care and time-sensitive clinical workflows.

## 3. Trends in X-Ray Imaging

X-ray imaging has undergone significant advancements in recent years, particularly with the integration of deep learning techniques. These developments have improved image quality, reduced noise, and enhanced diagnostic accuracy. In this section, we explore the latest trends shaping X-ray imaging, including the role of deep learning models in medical imaging, recent research contributions, and existing challenges. First, we provide an overview of X-ray imaging and its intersection with deep learning. Then, we review related works that highlight state-of-the-art methodologies. Finally, we discuss key research gaps and open challenges that need to be addressed to further advance this field.

### 3.1. Overview of X-Ray Imaging and Deep Learning

In cardiology, chest X-rays reveal critical signs such as cardiomegaly, pulmonary edema, and abnormal vascular patterns indicative of heart failure, coronary artery disease, and valvular disorders [76,77,78,79]. While lacking the functional insights of echocardiography or MRI, X-rays offer rapid, accessible preliminary assessments. Deep learning has enhanced X-ray diagnostics by enabling early detection of subtle indicators like minor cardiac enlargement and reducing inter-radiologist variability.

In this section, we use a heart dataset to demonstrate the challenges of deep learning. This dataset contains chest X-ray images taken for routine check-ups or to diagnose cardiovascular and pulmonary conditions [80]. The images were standardized to a uniform resolution and annotated by three radiologists, who segmented the lungs and heart. The dataset includes 114 images, each with three expert annotations, totaling 342 segmentations. The dataset is divided into training and testing subsets to support model development and evaluation. It serves as a valuable resource for medical image analysis, particularly for heart and lung segmentation tasks. In this context, the same deep learning model described in Section 2.1, enhanced with Bayesian optimization, is utilized. The results reveal a slight deviation in the predictions, where the ground truth labels are somewhat distant from the ROIs segmented by doctors. This discrepancy can be attributed to the correlational nature of the X-ray images, which introduces challenges in precise segmentation. Additionally, there may be limitations when compared to ultrasound images, as the results demonstrate poorer performance. However, the approach may still prove useful for certain applications.

**Figure 7:**
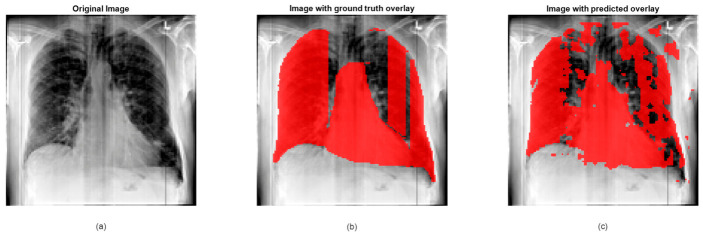
Example of X-ray image and deep learning-based segmentation: (**a**) Original chest X-ray image; (**b**) original image with overlaid segmentation mask highlighting ROIs; (**c**) predicted segmentation mask generated by the deep learning model, demonstrating close alignment with the ground truth.

The clinical relevance of these segmentation discrepancies cannot be overstated. In X-ray imaging, accurate delineation of the cardiac silhouette is essential for detecting cardiomegaly, a key indicator of heart failure, valvular disease, and cardiomyopathy. The segmentation errors observed in Figure 7c, where the predicted mask deviates from expert annotations, could lead to incorrect cardiothoracic ratio measurements. This may result in false positives, prompting unnecessary echocardiograms and patient anxiety, or false negatives, delaying diagnosis and treatment of underlying cardiac conditions. Furthermore, inaccurate heart segmentation can compromise automated triage systems that prioritize abnormal cases, potentially impacting patient management in high-volume clinical settings. These implications highlight that improving segmentation precision is not merely a technical pursuit but a clinical necessity for reliable AI-assisted diagnosis.

### 3.2. Related Works of X-Ray Imaging and Deep Learning

The state-of-the-art has achieved excellent advancements in the field of X-ray imaging and deep learning. For instance, the authors in [43] investigate a multimodal deep learning approach for risk assessment in patients with Ischemic Heart Disease (IHD) using ECGs and Chest X-rays (CXRs). The study utilizes a dataset of 2107 patients who underwent elective Percutaneous Coronary Intervention (PCI) at The University of Tokyo Hospital. The methods involve Deep Neural Network (DNN) models trained to detect Left Ventricular Systolic Dysfunction (LVSD) from 12-lead ECGs and cardiomegaly from CXRs. These models classify patients into four risk groups based on their ECG and CXR outputs. The study employs a multimodal paradigm by integrating ECG and CXR data for improved risk prediction. The primary heart conditions addressed include IHD, LVSD, and cardiomegaly. The results indicate that patients in the highest risk category (dual-modality high-risk group) had a significantly increased likelihood of Major Adverse Cardiovascular Events (MACEs), with a hazard ratio of 2.370 (*p* < 0.001) compared to the no-risk group.

In ref. [44], the authors explore the use of deep learning for diagnosing heart failure from chest X-ray images. They utilize 952 images from the National Institutes of Health (NIH) ChestX-ray8 dataset, which were relabeled by cardiologists into normal and heart failure categories. The study employs a CNN model using VGG16 with transfer learning architecture pre-trained on ImageNet. Data augmentation techniques, including scaling and rotation, were applied to improve model performance. Grad-CAM was used to enhance interpretability by visualizing important regions influencing classification decisions. The primary heart disease studied is heart failure, defined by cardiomegaly or pulmonary congestion. The model achieved an accuracy of 82%, with a sensitivity of 75% and specificity of 94.4%.

The authors in [45] present a deep learning approach for analyzing chest X-rays to predict cardiac events in heart failure patients. The study uses a dataset of 192 hospitalized heart failure patients from a single center, with chest X-ray data analyzed at admission and pre-discharge. The method employs a previously trained model based on ResNet-50 architecture to predict elevated Pulmonary Artery Wedge Pressure (PAWP), utilizing gradient-weighted class activation mapping (Grad-CAM) for visualization. The study follows an explainability paradigm by integrating tuned ResNet-50 predictions with conventional clinical prognostic factors. The primary heart conditions addressed include HFrEF and Heart Failure with Preserved Ejection Fraction (HFpEF). The model achieved an AUROC of 0.77 for predicting elevated PAWP, and its probability predictions significantly improved risk stratification for heart failure-related readmission and cardiac mortality.

The authors in [46] proposed a Multiple Tasking Wasserstein Generative adversarial network U-shape Network (MWG-UNet), a hybrid deep learning framework for lung and heart segmentation in chest X-ray images. The study uses two datasets: the Japanese Society of Radiological Technology (JSRT) dataset and the Shenzhen Hospital dataset, comprising a total of 862 images (340 normal and 275 abnormal X-rays). The method employs MWG-UNet, which integrates a generative model with a modified UNet that incorporates squeeze-and-excitation (SE) blocks for improved segmentation accuracy. The study follows generative adversarial learning and multi-task learning paradigms, focusing on lung field and heart segmentation for potential disease diagnosis. The results indicate strong segmentation performance, achieving a DSC of 95.28%, precision of 96.41%, and F1-score of 95.90% for lung segmentation. However, for heart segmentation, the model attained a Dice similarity of 71.16% and an IoU of 74.56%, suggesting room for improvement in multi-organ segmentation.

The authors in [47] present a deep learning approach for predicting survival in heart failure patients using CXRs. The study utilizes a dataset of 353 heart failure patients who underwent chest radiographs at The First Affiliated Hospital of Nanjing Medical University between 2012 and 2017. The methodology includes the development of Deep Learning Survival Prediction models using Chest Radiographs (DLSPCR), a CNN model with preprocessing techniques such as image resizing, normalization, and data augmentation. Additionally, a more advanced model integrates clinical factors using an MLP for enhanced risk stratification. The study follows an explainability paradigm, employing Grad-CAM to highlight significant regions in the CXRs. The primary disease addressed is heart failure, including cardiomegaly. The model achieved the highest performance, with AUROC values of 0.818 (5-year survival prediction), 0.702 (3-year), and 0.706 (1-year), surpassing traditional clinical and imaging models.

In ref. [48], the authors developed a deep learning approach for diagnosing multiple heart disease types using chest X-rays. The study utilizes a NIH Chest X-ray dataset containing 112,120 frontal-view X-ray images from 30,805 patients. The method employs a CNN with VGG16 architecture, leveraging data augmentation (rotation, width/height shifts, horizontal flips) and normalization to enhance feature extraction. The study follows a transfer learning paradigm, utilizing a pre-trained model on ImageNet. The heart diseases addressed include cardiomegaly, emphysema, effusion, hernia, infiltration, mass, nodule, atelectasis, pneumothorax, pleural thickening, pneumonia, fibrosis, edema, and consolidation. The model achieved an overall accuracy of 92.6% with a sensitivity of 92.9%, and disease-specific accuracies ranging from 82.96% (infiltration) to 95.37% (emphysema).

The authors in [49] investigate the use of deep learning algorithms to detect heart failure from chest X-ray images. The study utilizes a NIH X-ray dataset that contains 112,120 X-ray images with multiple labeled thoracic diseases, including cardiomegaly, which is considered an indicator of heart failure. The methodology involves testing twelve different CNN architectures, including Xception, DenseNet121, DenseNet169, MobileNetV2, EfficientNetB3, EfficientNetB0, ResNet-50, VGG16, NASNet-Mobile, Inception-V3, Vanilla CNN, and VGG19. The study employs a transfer learning paradigm, using pre-trained models trained on ImageNet and fine-tuned for heart failure detection. Among the tested architectures, the Xception model achieved the best performance, with an accuracy of 86%, a precision of 77.5%, a recall of 82%, and an F1-score of 79%. The results highlight the potential of deep learning models in automated heart failure diagnosis from chest radiographs.

The authors in [50] presented a model-based deep learning approach for heart segmentation in posterior–anterior (PA) chest X-ray images. The study utilizes a Japanese Society of Radiological Technology (JSRT) dataset (247 analog PA chest X-rays) and a private dataset (22 clinical images) for evaluation. The proposed method integrates Active Shape Models (ASM), deep CNN, and numerical optimization using the Alternating Direction Method of Multipliers (ADMM). The segmentation process includes corner point localization via a neural network, initialization of landmark points, iterative refinement using neural predictions, and post-processing with Catmull-Rom splines. The study employs a hybrid paradigm combining deep learning with model-based segmentation to improve accuracy and robustness, especially in challenging cases where lesions obscure heart boundaries. The primary cardiovascular diseases addressed include cardiomegaly, pleural effusion, and pulmonary edema. The proposed method achieved a Dice similarity score of 0.877 ± 0.05 and a Hausdorff distance of 17.35 mm ± 4.52 mm on the private dataset, demonstrating better generalization compared to purely neural network-based segmentation approaches.

Table 4 below summarizes this work for clearer and better-extracted details of previous reviews and analyses. Many X-ray-based deep learning studies do not explore advanced paradigms such as federated learning, uncertainty quantification, or reinforcement learning, while CNNs dominate and transformers, GANs, and autoencoders remain underutilized. Latency and computational efficiency remain critical, as large-scale models require substantial resources, limiting deployment in emergency or low-resource settings. Further efforts are needed to streamline architectures and optimize inference speed for real-time clinical workflows.

### 3.3. Discussion Research Gaps in X-Ray Imaging and Deep Learning

Despite significant advancements in deep learning applications for heart disease detection using X-ray imaging, several key gaps remain that warrant further exploration. One major challenge is the optimization of hyperparameters, such as learning rates, batch sizes, and network architectures, which are often tuned empirically rather than systematically. Many studies rely on standard architectures like VGG16, ResNet-50, and UNet, without extensive hyperparameter tuning tailored to medical imaging. Additionally, uncertainty quantification in model predictions is often overlooked, despite its critical importance in clinical decision-making. Methods such as Bayesian deep learning and Monte Carlo dropout could be integrated to provide confidence estimates for automated diagnoses. Furthermore, while traditional CNNs and attention-based mechanisms like Grad-CAM have improved interpretability, newer transformer-based architectures such as Vision Transformers and Swin Transformers offer superior global context awareness and adaptive feature extraction yet remain underutilized in heart disease imaging. Another promising yet underexplored area is the use of adaptive learning units, which dynamically adjust model complexity based on input variations, potentially improving generalizability across diverse datasets. Additionally, generative models like diffusion models and generative adversarial networks could enhance data augmentation, synthetic dataset creation, and anomaly detection, addressing challenges related to data scarcity and class imbalance. Future research should focus on integrating these cutting-edge algorithms with robust optimization strategies and uncertainty-aware frameworks to develop more reliable and interpretable deep learning models for medical imaging.

When comparing the results across these studies, several patterns emerge regarding accuracy, clinical applicability, and methodological diversity. Early works such as [48] achieved high classification accuracy (92.6%) on NIH Chest X-ray images using transfer learning with VGG16, demonstrating the potential of deep CNNs for cardiomegaly detection. More recent studies, such as [49], validated multiple architectures on large-scale datasets, with Xception achieving 86% accuracy and balanced performance across precision, recall, and F1-score, reflecting progress toward more reliable benchmarks. In contrast, ref. [44] reported similar accuracy levels of around 82% when predicting heart failure conditions, with relatively high specificity (94.4%) but lower sensitivity, highlighting the trade-offs between detection robustness and clinical utility. Multimodal approaches like [43], which combined ECG and X-ray data, produced AUROC values of 0.77 for PAWP prediction, underscoring the potential of integrating complementary modalities for risk stratification. Segmentation-focused works ([46]) reached Dice scores above 0.87, with MWG-UNet achieving particularly strong performance for lung regions (95.28%), though heart segmentation proved more challenging with lower Dice values (~71%). Overall, classification studies often reported accuracies exceeding 80–90%, but external generalizability remained variable, while segmentation works showed that hybrid and generative approaches can boost structural delineation. Taken together, these results suggest that although CNN-based models consistently outperform traditional methods, their performance is heavily influenced by dataset scale, task type, and methodological design. This highlights the need for more standardized evaluations across diverse populations, as well as greater exploration of advanced architectures such as transformers and hybrid generative-discriminative frameworks to push the boundaries of diagnostic accuracy and robustness in X-ray imaging for cardiovascular disease.

Although deep learning has significantly advanced the use of chest X-rays for heart disease detection, latency and deployment limitations remain important concerns. Many classification studies, such as those using VGG16, ResNet-50, or Xception [44], reported strong accuracy but relied on deep CNNs with substantial computational costs, which could hinder real-time applicability in clinical or low-resource settings. Segmentation-focused frameworks like MWG-UNet [46] and ASM-CNN-ADMM [50] achieved robust Dice scores but incorporated generative and hybrid optimization strategies that increase inference time and memory requirements, making them difficult to run efficiently on portable or embedded systems. Multimodal approaches combining CXR and ECG data [43] introduced additional preprocessing and synchronization steps, further contributing to latency and limiting bedside usability. Moreover, while Grad-CAM-based interpretability enhanced trust, it also added computational overhead during inference. These trade-offs between accuracy, interpretability, and efficiency underscore the need for streamlined architectures, lightweight model compression techniques, and deployment-focused optimization. Without addressing these challenges, deep learning for X-ray imaging risks remaining confined to research settings rather than being translated into time-sensitive clinical workflows.

## 4. Trends in MRI and Deep Learning

This section is an overview with examples of MRI and deep learning, and an analysis of related works. It starts by providing a broad definition of MRI, underscoring its importance in medical diagnostics, especially for cardiac applications. The section then explores the challenges inherent to MRI, such as variations in image quality, the presence of artifacts, and the intricacies of analyzing complex, multi-dimensional data. Practical examples are presented using real-world datasets, illustrating how deep learning methods can improve the interpretation of MRI images, often revealing intricate details that are not easily detectable through traditional methods. The section also includes a thorough review of related works, discussing existing research at the intersection of MRI and deep learning, identifying gaps in the current literature, and shedding light on emerging trends and future directions in the field.

### 4.1. Overview of MRI and Deep Learning

In cardiac imaging, MRI provides a detailed assessment of myocardial tissue, heart chambers, and blood flow, making it invaluable for diagnosing myocardial infarction, cardiomyopathies, and congenital heart defects [81,82,83]. Unlike ultrasound, MRI excels in visualizing deeper structures and quantifying tissue properties. Deep learning has further advanced cardiac MRI by enabling faster analysis and automated detection of subtle abnormalities.

For illustration, we have used the Automatic Cardiac Diagnosis Challenge (ACDC) dataset, which is a comprehensive collection of cardiac MRI images designed to facilitate research in automated cardiac diagnosis [84]. The dataset includes multi-center, multi-vendor, and multi-disease cases, making it highly diverse and representative of real-world clinical scenarios. It comprises short-axis cardiac MRI scans from 150 patients, with each patient’s data including end-diastolic and end-systolic frames, as well as manual annotations of the left ventricle, right ventricle, and myocardium. These annotations are provided by clinical experts, ensuring high-quality ground truth for training and evaluation. The ACDC dataset is particularly valuable for tasks such as image segmentation, cardiac volume estimation, and disease classification. It includes cases of five distinct cardiac pathologies: dilated cardiomyopathy, hypertrophic cardiomyopathy, myocardial infarction, abnormal right ventricle, and normal hearts. This variety allows researchers to develop and test algorithms that can generalize across different conditions. Additionally, the dataset provides clinical parameters such as ejection fraction, which is crucial for assessing cardiac function. By offering a standardized benchmark, the ACDC dataset has become a key resource for advancing the field of medical image analysis and machine learning in cardiology.

**Figure 8:**
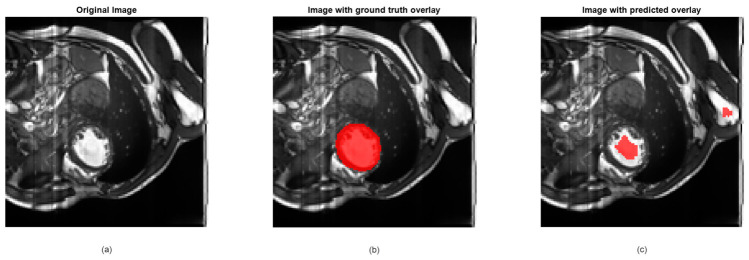
Example of cardiac MRI segmentation: (**a**) Original slice; (**b**) ground truth mask; (**c**) predicted mask with deep learning.

Figure 8 presents a comparative visualization of some important illustrative results obtained from the cardiac MRI segmentation process using the ACDC dataset. This figure was generated using the same deep learning model described in Section 2.1, along with the same optimization procedures involving Bayesian optimization. The goal is to visually assess which types of representations are more accurate in addressing the challenges faced by deep learning models in the context of MRI image segmentation, including generalization capabilities and comparisons with US imaging. The figure is divided into three subplots, each highlighting a different aspect of the segmentation task. In Figure 8a, the original grayscale cardiac MRI image is displayed. This image serves as the baseline input for the segmentation process, showcasing the anatomical structures of the heart, including the left ventricle, right ventricle, and myocardium. The image is normalized to enhance contrast and visibility, providing a clear representation of the cardiac region of interest. Figure 8b overlays the ground truth mask onto the original image. The ground truth mask, manually annotated by clinical experts, is displayed in red with a transparency level of 70%. This overlay allows for a direct visual comparison between the original image and the expert-annotated regions, highlighting the precise boundaries of the cardiac structures. The ground truth mask is crucial for evaluating the accuracy of automated segmentation methods. Figure 8c shows the predicted segmentation mask overlaid on the original image. The predicted mask, generated by the CNN model trained on the ACDC dataset, is also displayed in red with 70% transparency. This subplot demonstrates the performance of the CNN in segmenting the cardiac structures. The predicted mask is thresholded at 0.6, the same as in the previous example in Section 2.1, to create a binary output, which is then compared to the ground truth mask to assess the model’s accuracy. The predicted mask appears to capture regions of interest but also includes areas that are far from the actual regions of interest. One commonality between the predicted mask and the ground truth may be the color intensity of the pixels. In this context, we observed a challenging situation that could potentially lead to errors when compared to the ground truth. This misprediction is likely due to the complexity of the cardiac structures, variations in image quality, or limitations in the model’s ability to generalize across diverse cases.

Additionally, compared to US images, the predicted mask aligns more closely with the object of interest, similar to the ground truth, with only minor fluctuations. This suggests that the model performs more reliably on US data than on MRI images, possibly due to the higher resolution and more anatomical boundaries in MRI. This observation justifies the use of MRI and US for cardiac segmentation tasks and highlights the need for further refinement of the model when applied to both. Overall, Figure 8 provides a comprehensive visual comparison of the original image, ground truth mask, and predicted segmentation mask, illustrating the effectiveness of the CNN in automating cardiac MRI segmentation. The figure underscores the potential of deep learning techniques in medical image analysis while also highlighting areas for improvement in achieving closer alignment with expert annotations.

The clinical implications of these segmentation inaccuracies are substantial. In cardiac MRI, precise delineation of the left ventricle, right ventricle, and myocardium is fundamental for calculating clinical indices such as ejection fraction, ventricular volumes, and myocardial mass, which guide diagnosis and treatment in conditions like dilated cardiomyopathy, hypertrophic cardiomyopathy, and myocardial infarction. The extraneous regions included in the predicted mask shown in Figure 8c could lead to overestimation of ventricular volumes, potentially misclassifying a patient’s disease severity or treatment response. For example, overestimated left ventricular volumes might suggest more advanced heart failure than actually present, leading to unnecessary intensification of therapy or inappropriate device implantation. Conversely, underestimated volumes could mask disease progression, delaying critical interventions. These potential consequences underscore that achieving precise, reliable segmentation is not merely an algorithmic goal but a clinical imperative for safe and effective integration of deep learning into cardiovascular care.

### 4.2. Related Works of MRI and Deep Learning

Recent advancements in state-of-the-art research have highlighted the transformative potential of deep learning in the field of cardiac MRI. For instance, in ref. [51], the authors utilize two datasets for their study: the Medical Image Computing and Computer-Assisted Intervention (MICCAI) 2012 Right Ventricle (RV) segmentation challenge database and the MICCAI 2009 Left Ventricle (LV) database. The methods applied involve a three-stage deep learning approach: (1) automatic localization of the heart chamber using CNNs, (2) initial segmentation using a stacked autoencoder, and (3) refinement of the segmentation using deformable models. This approach is inspired by human brain function to achieve accurate, fast, and robust segmentation of RV and LV from cardiac MRI datasets. The primary focus is on segmenting heart chambers (RV and LV) for evaluating cardiac function, rather than diagnosing specific heart diseases. The accuracy metrics reported include a DSC of 0.81 for RV segmentation, and high correlation coefficients (0.99) for LV End-Diastolic Volume (EDV), End-Systolic Volume (ESV), and ejection fraction (EF), demonstrating the method’s high accuracy and clinical applicability.

In the study developed in [52], the authors utilize the MICCAI 2009 LV segmentation challenge database, which includes 45 cardiac Short-Axis (SAX) Cine-MR datasets divided into training, testing, and online sets. The methods applied involve a combination of deep learning (using Deep Belief Networks, DBNs) and level set methods for the automated segmentation of the left ventricle’s endocardial and epicardial borders from cardiac cine-MR images. The process includes three main steps: (1) ROI detection using DBNs, (2) initial segmentation using Otsu’s thresholding, and (3) refinement using a Distance-Regularized Level Set (DRLS) method. The primary focus is on segmenting the left ventricle for evaluating cardiac function, rather than diagnosing specific heart diseases. The accuracy metrics reported include DSC and Average Perpendicular Distance (APD), with the proposed method achieving a DSC of 0.91 for endocardium segmentation and 0.94 for epicardium segmentation, demonstrating high accuracy and clinical applicability.

In ref. [53], the authors utilize three datasets for their study: the ACDC dataset, the Medical Segmentation Decathlon Task02 Heart dataset (MSD02), and the Myocardial Pathology Segmentation Combining Multi-sequence CMR dataset (MyoPS 2020). The ACDC dataset, which includes 300 cine-MRIs from 150 patients, is primarily used for evaluation and visualization, while the MSD02 and MyoPS 2020 datasets are employed to test the adaptability of the proposed method across different datasets. The study focuses on developing nn-TransUNet, an automatic deep learning pipeline for heart MRI segmentation, which combines the experiment planning of nnUNet with the network architecture of TransUNet. The pipeline employs vision transformers and convolutional layers in the encoder and uses convolutional layers as the decoder. The method involves adaptive preprocessing, network training, and automatic experiment planning to optimize segmentation tasks without manual parameter tuning. The heart diseases addressed include conditions related to the LV, RV, and myocardium, as well as myocardial edema and scars. The accuracy of the segmentation is evaluated using metrics such as DSC, Hausdorff Distance (HD), and Jaccard Similarity Coefficient (JSC), with nn-TransUNet achieving state-of-the-art performance on the ACDC dataset, demonstrating high accuracy in segmentation tasks.

The authors in [54] investigated a dataset consisting of 44 patients (24 males and 20 females; mean age, 41.3 years) with cerebral disorders who underwent both contrast transcranial Doppler sonography (cTCD) and non-enhanced cardiac MRI (CMRI) examinations. Additionally, data from 335 patients were collected by 2023. The study employs deep learning to analyze CMRI cine images obtained using a 3.0T MR scanner with the OBL FIESTA CINE 4CH sequence. The images were processed using AW station 4.7, and pseudo-color coding was applied to observe blood shunt conditions, measure the length and width of Patent Foramen Ovale (PFO), and identify complications such as Interatrial Septum (IAS) aneurysm or secondary septum thickening. The study does not explicitly mention the use of advanced paradigms like transfer learning, federated learning, reinforcement learning, explainability, interpretability, or uncertainty quantification. The primary heart condition addressed is PFO, with specific attention to its classification and associated complications like IAS aneurysm and septum thickening. The accuracy metrics reported include a sensitivity of 94.9%, specificity of 60.0%, accuracy of 90.9%, positive predictive value of 94.9%, negative predictive value of 60.0%, and an AUROC of 0.774, demonstrating the diagnostic value of CMRI with deep learning for PFO assessment.

In ref. [55], the authors utilize the UK Biobank dataset, which includes CMR imaging data from over 500,000 participants aged 40–69 years. The study focuses on 4723 individuals with Cardiovascular Disease (CVD) and 5733 healthy controls. The CMR images were acquired using a 1.5 Tesla scanner, with short- and long-axis cine images analyzed automatically using the Circle cvi42 software, which employs a CNN for segmentation. The study adapts a 3D UNet (LC-UNet) to predict dense 3D segmentations of the heart chambers (left and right ventricles and atria) from sparse 2D slices, enabling more accurate volume estimation. The authors do not explicitly mention the use of advanced paradigms such as transfer learning, federated learning, reinforcement learning, explainability, interpretability, or uncertainty quantification. The heart diseases addressed include heart failure, Myocardial Infarction or Ischemic Heart Disease (MI-IHD), Ventricular Arrhythmia (VAC), Conduction Defects (CD), and Atrial Fibrillation (AF). The accuracy metrics reported include univariate and multivariate regression analyses, with the deep learning method showing stronger associations with cardiovascular risk factors and disease compared to standard volume estimation methods. The AUROC for logistic regressions was higher for deep learning volumes (e.g., 0.918 for LV EDV) than for standard volumes (e.g., 0.902 for LV EDV), indicating improved diagnostic performance.

Table 5 summarizes clearly the results of related research on MRI and deep learning for heart disease detection. Many MRI-based deep learning studies do not explore advanced paradigms such as transfer learning, explainability, federated learning, or uncertainty quantification, while CNNs and UNets dominate and transformers, GANs, and autoencoders remain underutilized. Large-scale models and multi-step pipelines raise concerns about computational efficiency and real-time feasibility, limiting deployment in clinical settings. Further efforts are needed to streamline architectures, improve generalization across diverse datasets, and validate models on low-power platforms.

### 4.3. Discussion of Research Gaps in MRI and Deep Learning

The studies summarized in Table 5 reveal several research gaps in the application of MRI and deep learning for heart disease detection. Notably, these works do not explore advanced research paradigms such as transfer learning, explainability, or interpretability, which could enhance model generalizability and transparency. Additionally, there is a lack of utilization of reinforcement learning and federated learning, which could improve adaptive learning capabilities and enable collaborative, privacy-preserving model training across institutions. In terms of methods, advanced techniques like GANs, transformers, and autoencoders are relatively scarce, despite their potential to improve feature extraction, data augmentation, and representation learning. Furthermore, adaptive deep learning mechanisms, such as attention mechanisms, variational models, and recurrent architectures like LSTMs, are underutilized, representing a significant gap in leveraging temporal and contextual information for more accurate and dynamic heart disease detection. The accuracy metrics, while promising, show variability across studies. These variations highlight the need for more robust and consistent models. Therefore, future research should focus on integrating advanced paradigms and techniques, including adaptive deep learners, to address these gaps, improve accuracy, and enhance the overall reliability and interpretability of deep learning models in cardiac MRI analysis.

When comparing these MRI-based studies, it is clear that both dataset scale and methodological design play critical roles in performance outcomes. Earlier works such as [51] focused on LV and RV segmentation using CNNs, autoencoders, and DBNs, reporting Dice scores between 0.81 and 0.94, with particularly high agreement for endocardium and epicardium segmentation. More recent approaches, such as [53] with nn-TransUNet, demonstrated notable improvements, achieving a DSC of 93.6% and a Jaccard score of 88.3% on multi-dataset benchmarks, showing the potential of transformer-based models for cardiac MRI. Clinical diagnostic applications were explored in [54], where deep learning achieved high sensitivity (94.9%) and overall accuracy (90.9%) for detecting PFO and associated complications, though specificity remained modest (60.0%), indicating challenges in avoiding false positives. Large-scale population studies such as [55], leveraging the UK Biobank cohort of over 500,000 participants, reported AUROC values up to 0.918 for LV functional estimation, underscoring the scalability and prognostic value of 3D UNet-based models in linking imaging phenotypes with cardiovascular outcomes. Collectively, these results illustrate a trajectory from traditional segmentation-focused methods toward clinically integrated, large-scale predictive frameworks. While segmentation accuracy has reached high levels across benchmark datasets, generalization to diverse populations and broader disease categories remains variable. This comparison emphasizes the need for advanced paradigms, such as explainability, federated learning, and generative or transformer-based architectures, to bridge the gap between technical performance and clinical adoption in MRI-based heart disease detection.

Despite the strong performance of deep learning in cardiac MRI analysis, latency and methodological limitations remain important barriers to clinical translation. Early CNN and autoencoder-based approaches [51] achieved moderate to high segmentation accuracy, yet required multi-step refinement processes that introduced additional inference time, limiting their practicality for real-time applications. More recent transformer-based models such as nn-TransUNet [53] demonstrated state-of-the-art Dice scores, but their reliance on complex encoder–decoder architectures with self-attention layers significantly increases memory and computational demands, making deployment on resource-constrained MRI workstations challenging. Clinical diagnostic applications, such as PFO detection with CMRI [54], reported high sensitivity but relatively low specificity, highlighting the trade-off between diagnostic accuracy and generalizability, which can delay clinical decision-making if models generate excessive false positives requiring manual review. Population-scale applications, such as large cohort analyses using 3D UNet architectures [55], provided strong predictive value but necessitated extensive preprocessing of volumetric data, resulting in long pipeline runtimes that may be impractical in acute care or time-sensitive scenarios. Collectively, these limitations underscore the tension between accuracy and efficiency in MRI-based deep learning pipelines, suggesting that future progress will depend on integrating model compression, accelerated inference strategies, and lightweight architectures capable of balancing diagnostic precision with clinical feasibility.

## 5. Trends in Computed Tomography Imaging

Computed tomography imaging has seen significant advancements with the integration of deep learning techniques, enhancing its accuracy and applicability across various fields. This section explores recent trends in computed tomography, focusing on how deep learning has contributed to its evolution. It begins with an overview of the technology and its synergy with artificial intelligence, followed by a review of related works that have leveraged deep learning for improved cardiac image analysis. Finally, the discussion highlights existing research gaps, identifying opportunities for further development in this rapidly growing field.

### 5.1. Overview of Computed Tomography and Deep Learning

In cardiac imaging, CT is essential for evaluating coronary artery disease, assessing cardiac anatomy, and planning interventions such as transcatheter aortic valve implantation [85]. Unlike echocardiography, CT excels in providing high-resolution anatomical detail, enabling detection of stenosis, plaque burden, and anatomical variations. Deep learning has further enhanced cardiac CT by automating segmentation and plaque quantification.

**Figure 9:**
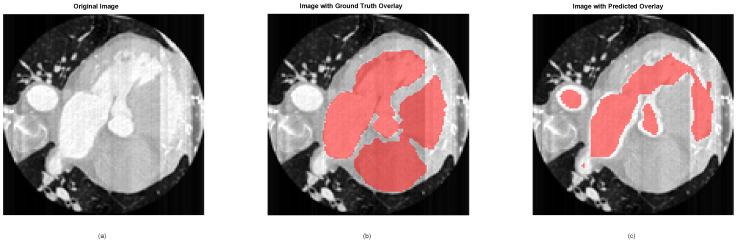
Example of cardiac CT image segmentation: (**a**) Original slice; (**b**) ground truth mask; (**c**) predicted mask with deep learning.

For this study and illustration purposes, we have used the Multimodality Whole-Heart Segmentation (MM-WHS) 2017 dataset. This dataset is designed for the automatic segmentation of cardiac structures using CT and MRI [86]. It includes 60 whole-heart scans (40 for testing, 20 for training) with expert-labeled annotations of key cardiac structures, such as the LV, RV, Left Atrium (LA), Right Atrium (RA), Myocardium (Myo), Aorta (Ao), and Pulmonary Artery (PA). The dataset serves as a valuable benchmark for developing and evaluating machine learning and deep learning models in medical image segmentation and cardiac imaging research. Figure 9 in this context is generated using the same approach as previous experiments (Section 2.1), employing a small CNN architecture. The key difference is that we use 20 labeled CT images from the MM-WHS dataset, 19 for training and one for plotting this figure. In particular, Figure 9a represents the original image, while Figure 9b shows the segmentation ground truth, with a red overlay mask defined by expert annotations. Figure 9c presents the segmentation output obtained from our deep learning model. From the authors’ perspective and based on visual evaluation, the CT images exhibit better segmentation performance compared to previous US, MRI, and X-ray images. This may be due to the higher contrast and structural clarity of CT scans, which facilitate the identification of segmented regions more accurately.

However, some jittered regions and incorrect segmentations are present, highlighting several challenges in the segmentation process. This due to the fact that deep learning-based CT segmentation of the heart presents several challenges that impact accuracy and reliability. Noise and artifacts, such as motion blur, beam hardening, and scanner-induced distortions, can degrade image quality and mislead the model during training and inference. Class imbalance is another major issue, as certain anatomical structures may be underrepresented in the dataset, leading to biased predictions and poor generalization. Boundary ambiguity further complicates segmentation, as overlapping tissues, low contrast regions, and unclear edges make it difficult to define precise anatomical structures. The problem is exacerbated by variability in annotation, where expert-defined ground truths may differ due to subjective interpretation, introducing uncertainty into model training. Lastly, model generalization can be difficult to achieve, as variations in scanning protocols, patient anatomy, and imaging conditions can cause the model to struggle with unseen data. Overcoming these challenges requires a combination of data augmentation techniques, advanced model architectures, domain adaptation strategies, and the use of larger, more diverse datasets to enhance segmentation performance and robustness.

These technical challenges translate directly into clinical risks. In cardiac CT, accurate segmentation of cardiac structures is essential for applications such as coronary artery disease assessment, surgical planning for transcatheter aortic valve implantation, and quantification of atrial and ventricular volumes. The jittered regions and incorrect segmentations observed in Figure 9c could lead to clinically significant errors. For instance, inaccurate delineation of the left atrial appendage might compromise stroke risk assessment in atrial fibrillation patients, while errors in aortic root segmentation could affect prosthetic valve sizing during TAVI planning, potentially leading to procedural complications such as paravalvular leakage or device mismatch. Similarly, misclassification of myocardial boundaries may impact viability assessment in ischemic heart disease. These potential consequences demonstrate that addressing the technical challenges of CT segmentation (e.g., noise, class imbalance, boundary ambiguity, and annotation variability) is not merely an academic exercise but a prerequisite for ensuring patient safety and treatment efficacy in CT-guided cardiovascular interventions.

### 5.2. Related Works of Computed Tomography and Deep Learning

A set of important works has been carried out in the context of CT and deep learning. Among them, we review a fresh list as described in Table 2. The authors in [56] developed a deep learning system for volumetric heart segmentation to improve treatment planning in radiation oncology, particularly for breast cancer patients. The dataset used includes multi-center data of 858 cases for training and an independent validation set of 5677 breast cancer patients treated at Dana-Farber/Brigham and Women’s Cancer Center. The methods involve deep learning models based on a 3D UNet architecture, trained using expert-labeled ECG-gated and low-dose chest CT scans. The study applies the paradigm of domain transfer, where an AI system initially trained in cardiovascular radiology is repurposed for radiation oncology. The primary heart diseases considered are radiation-induced cardiac complications such as coronary artery disease and heart failure. Reported accuracy metrics include a DSC of 0.95 for training data, 0.92 for expert-assisted segmentation, and 0.89 in real-world clinical applications, demonstrating the system’s effectiveness in reducing segmentation time while maintaining high concordance.

The work in [57] presents a deep learning-based method for whole-heart segmentation in 4D contrast-enhanced cardiac CT images, particularly for patients undergoing Transcatheter Aortic Valve Implantation (TAVI). The study dataset comprises 4D contrast-enhanced cardiac CT scans from 1509 patients, with 21,605 3D images divided into a development set (12 patients) and a test set (1497 patients). The method employs a 3D CNN with an encoding-decoding structure, utilizing residual blocks and batch normalization for automatic multi-class segmentation of cardiac structures, including the LV Myocardium, LV cavity, right ventricle, left atrium, and right atrium. The study incorporates cross-validation and ensemble learning to enhance robustness and generalization. The primary paradigm used in this research is deep learning with an emphasis on explainability and generalization across different cardiac phases. The paper focuses on cardiac diseases relevant to TAVI, such as aortic stenosis and other structural heart conditions. Accuracy metrics include a Dice similarity coefficient (DSC) of 0.89 ± 0.10 and an Average Symmetric Surface Distance (ASSD) of 1.43 ± 1.45 mm in 3D segmentations, with qualitative evaluation confirming clinically useful segmentations in over 90% of cases.

The authors in [58] proposed an efficient hybrid deep learning network model for the segmentation and classification of heart angiographic images. The dataset used consists of 400 Coronary Computed Tomography Angiography (CCTA) images collected from two hospitals in Peshawar, Pakistan. The study applies a multi-stage method: contrast enhancement using Contrast-Limited Adaptive Histogram Equalization (CLAHE), segmentation using a Modified UNet, feature extraction with the Self-Attention Network (SAT-Net), and classification via an Enhanced Elman Spike Neural Network (EESNN). Additionally, feature selection is optimized using the Red Fox Optimization (RFO) algorithm to improve classification accuracy. The study employs paradigms of deep learning, explainability, and optimization for improved interpretability and performance. The heart diseases addressed include different types of vascular abnormalities, such as blood flow-reduced vessels, narrow vessels, blocked vessels, and normal vessels. Reported accuracy metrics include a DSC and Intersection over Union (IoU), with high segmentation and classification accuracy achieved through the proposed framework.

The authors in [58] propose an automatic whole-heart segmentation method that combines deep learning with shape-context modeling to enhance accuracy. The dataset used is the MM-WHS challenge dataset, which includes 20 contrast-enhanced CT scans and 20 contrast-enhanced MRI scans with manually annotated ground truth. The method involves a three-step pipeline: scout segmentation using orthogonal 2D UNets, shape-context estimation using statistical shape models, and final refinement with a shape-context-guided UNet. The study integrates paradigms of deep learning and statistical shape modeling to improve interpretability and segmentation precision. The heart structures segmented include the left and right ventricles, left and right atria, left ventricular myocardium, ascending aorta, and pulmonary artery, which are relevant to various cardiac diseases. The reported accuracy metrics include DSC of up to 0.935 for the left ventricle in CT images and 0.895 for MRI images, demonstrating the effectiveness of integrating shape context with deep learning.

Table 6 summarizes in a clearer way the analysis of related works on computed tomography and deep learning for heart disease detection. Many CT-based deep learning studies do not explore advanced paradigms such as federated learning, explainability, or uncertainty quantification, while 3D UNet and CNNs dominate and transformers, GANs, and shape-context modeling remain underutilized. The computational complexity and resource-intensive nature of these models hinder real-time clinical applications and deployment on low-power platforms. Further efforts are needed to address shape variations, noise, and data heterogeneity while improving model generalizability across diverse datasets.

### 5.3. Discussion of Research Gaps in Computed Tomography and Deep Learning

Despite these advancements, several challenges remain in this domain. One of the key issues is the need for larger, more diverse datasets to enhance model generalization and reduce bias. Another challenge is the computational complexity and resource-intensive nature of deep learning models, which can hinder real-time clinical applications. Additionally, the integration of explainability and interpretability remains crucial for gaining trust in AI-driven medical diagnoses. Some studies have also reported difficulties in handling shape variations and noise in medical images, which affect segmentation accuracy. Future research should focus on addressing these challenges by incorporating techniques such as federated learning for data privacy, uncertainty quantification for model reliability, and improved data augmentation strategies to enhance robustness.

When comparing the CT-based studies, it becomes evident that progress has been driven by both methodological innovations and dataset diversity. The pioneering work of [56] applied a 3D UNet across multi-center datasets, achieving Dice scores as high as 0.95 in training and 0.89 in real-world deployment, highlighting the challenge of performance drop when models transition to clinical use. In ref. [57], a large cohort of 1509 patients with 4D CT scans enabled robust whole-heart segmentation using residual CNNs, reaching a DSC of 0.89, with relatively low average surface distance errors, demonstrating the feasibility of accurate volumetric analysis across cardiac phases. More recent approaches, such as ref. [58], integrated contrast enhancement, modified UNet, and ensemble feature selection to classify vascular conditions, reporting high segmentation accuracy and reliable vessel classification, thus underscoring the value of optimized hybrid pipelines. Meanwhile, ref. [59] advanced shape-context-guided UNet refinements, achieving DSC values up to 0.935 for left ventricle segmentation, showing the potential of combining statistical shape modeling with deep learning to capture complex cardiac structures. Collectively, these studies illustrate that while CT-based methods can achieve segmentation and classification metrics exceeding 0.9 in controlled or challenge-based settings, translation to heterogeneous clinical populations remains more variable. The comparison underscores both the maturity of deep learning for CT segmentation and the ongoing need for generalizable, explainable, and resource-efficient frameworks to ensure reliable deployment in diverse cardiac diagnostic workflows.

While CT-based deep learning approaches have achieved impressive segmentation and classification accuracy, latency and methodological limitations remain important considerations for clinical integration. Early works such as [56] demonstrated high Dice scores during training (0.95) but reported a notable performance drop in real-world deployment (0.89), reflecting the challenges of model generalization and the added inference time introduced by expert-assisted refinement steps. Similarly, large-scale CNN-based frameworks for whole-heart segmentation [57] achieved strong accuracy across cardiac phases, but the reliance on residual networks and volumetric processing significantly increases computational overhead, limiting feasibility for real-time or point-of-care use. Hybrid pipelines that integrate preprocessing, feature selection, and ensemble classifiers [58] achieved robust vessel classification but introduced multi-stage dependencies that extend runtime and complicate clinical deployment. Even advanced shape-context-guided UNets [59], while improving segmentation precision to Dice scores above 0.93, remain computationally demanding due to iterative refinement stages and statistical shape modeling, raising concerns about inference speed on standard clinical hardware. Collectively, these findings suggest that despite methodological sophistication, CT-based models still face latency bottlenecks and performance variability in real-world contexts, underscoring the need for streamlined architectures, model compression, and accelerated inference strategies to balance accuracy with clinical efficiency.

## 6. Trends in Electrocardiogram Imaging and Deep Learning

This section explores an overview of ECG imaging and its role in deep learning-based heart disease detection, discussing its significance in medical diagnostics. It reviews related works that have applied various deep learning techniques, such as CNNs and hybrid models, to classify and segment ECG signals for detecting abnormalities like arrhythmias, myocardial infarctions, and other cardiac conditions. Additionally, this section identifies existing gaps in research, including challenges related to dataset availability, model generalizability, computational limitations, and the need for improved interpretability in AI-driven ECG analysis. It highlights the importance of addressing these gaps to enhance the accuracy, efficiency, and clinical applicability of deep learning models in real-world cardiac healthcare.

### 6.1. Overview of Electrocardiogram Imaging and Deep Learning

While ECG is fundamentally a physiological signal, it is treated as an imaging modality in this review because deep learning studies frequently convert ECG recordings into image representations (e.g., scalograms, spectrograms, or photographed paper traces) for analysis using computer vision architectures [87,88]. For illustration purposes of deep learning challenges in ECG image segmentation, we take the following dataset: the ECG Image Dataset from the National Heart Foundation of Bangladesh [89]. This dataset includes ECG images categorized into four groups: abnormal heartbeat patients, myocardial infarction patients, normal persons, and patients with a history of myocardial infarction. The images capture cardiac electrical activity, aiding in diagnosing arrhythmias, myocardial infarctions, and other heart conditions. The dataset consists of digital images in various resolutions and formats (JPEG, PNG, or DICOM) and may include metadata for clinical context. It is useful for training machine learning models for automated ECG analysis, arrhythmia detection, and cardiac health monitoring, supporting researchers, clinicians, and educators.

**Figure 10:**
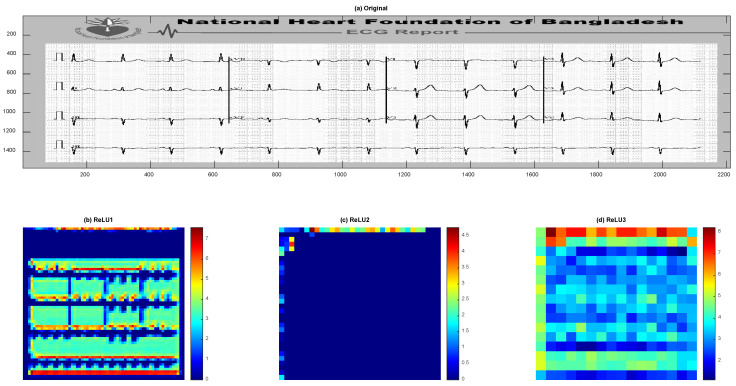
Different representations of ECGs at different deep layers of the custom CNN: (**a**) First input layer; (**b**–**d**) ReLU1, ReLU2, and ReLU3, respectively. Note: ECG is included as an imaging modality in this review because deep learning research commonly transforms ECG signals into image formats (e.g., scalograms, spectrograms) for analysis.

The implemented algorithm follows a structured deep learning pipeline for ECG image segmentation and classification. Initially, 20 images were selected from two different heart conditions: abnormal heartbeat patients and myocardial infarction patients. These images were then split into 60% for training and 40% for testing, solely for illustration purposes. ECG images are preprocessed, including grayscale conversion, resizing, normalization, and denoising using a pretrained DnCNN network [90]. Due to computational limitations, all images are resized to 256X256 to optimize processing efficiency while maintaining essential features. A custom CNN architecture is designed, comprising three convolutional layers (Conv1, Conv2, Conv3) with corresponding ReLU activation layers, max-pooling layers, and a fully connected classification layer. Bayesian optimization is employed to fine-tune hyperparameters, enhancing model performance. To visualize hidden layer activations, the model extracts feature maps from ReLU1, ReLU2, and ReLU3 layers as addressed by Figure 10. The original test image is displayed alongside its corresponding feature maps, illustrating how hierarchical feature representations evolve across deeper layers. This approach aids in understanding the CNN’s learned spatial patterns and segmentation effectiveness. White pixels vanish but patterns appear in white and become more localized. This process demonstrates how CNN models interpret ECGs in their unique way.

ECG analysis using CNNs presents several challenges, including a low signal-to-noise ratio due to artifacts from muscle activity and electrode movement, making feature extraction difficult. Unlike traditional images, ECGs are time-series signals with complex temporal dependencies, requiring models to capture both spatial and sequential patterns. Patient variability further complicates generalization, as ECG waveforms differ significantly based on age, health conditions, and genetic factors. Additionally, subtle abnormalities, such as arrhythmias, may be difficult to detect due to their small-scale waveform deviations. The scarcity of labeled ECG datasets and the interpretability issues of deep learning models further hinder the reliable deployment of CNNs in clinical practice.

These technical challenges carry profound clinical consequences. In ECG interpretation, accurate detection of subtle waveform deviations is essential for diagnosing life-threatening conditions such as myocardial infarction, life-threatening arrhythmias, and ischemic heart disease. The feature evolution shown in Figure 10 demonstrates how CNNs progressively focus on clinically relevant patterns, but the challenges of noise, patient variability, and small-scale abnormalities highlight why automated ECG analysis remains clinically challenging. For instance, failure to detect subtle ST-segment elevations could result in missed myocardial infarction diagnosis, delaying life-saving reperfusion therapy. Similarly, misclassification of arrhythmias such as atrial fibrillation might lead to inappropriate anticoagulation decisions, exposing patients to unnecessary bleeding risk or leaving them vulnerable to stroke. The scarcity of labeled datasets and interpretability issues further complicate clinical adoption, as clinicians must trust that model decisions align with established electrocardiographic criteria. These implications underscore that improving signal quality, model robustness, and interpretability is not merely a technical objective but a clinical necessity for reliable AI-assisted ECG diagnosis.

### 6.2. Related Works in Electrocardiogram Imaging and Deep Learning

According to the methodology of paper collection under the proposed data cleaning algorithm script, few studies have been conducted on ECG and deep learning in the context of heart disease detection.

The authors in [42] present a multi-center retrospective cohort study applying deep learning ECGs to detect left heart valvular dysfunction. The dataset consists of 617,338 ECGs paired with transthoracic echocardiograms (TTEs) from 123,096 patients across five hospitals in the Mount Sinai Health System in New York City, ensuring a diverse demographic representation. The study employs natural language processing (NLP) techniques to extract ground-truth labels from echocardiogram reports, which are then paired with ECG recordings for model training. A deep learning model combining an MLP and EfficientNet-based CNN is used to analyze ECG waveforms and detect valvular disease. The study follows paradigms of deep learning, interpretability, and external validation, ensuring model robustness across different patient populations. The primary heart diseases addressed are mitral regurgitation (MR) and aortic stenosis (AS), two common valvular disorders that pose significant health risks. The model achieves high performance, with AUROC scores of 0.88 (internal) and 0.81 (external) for MR detection, and 0.89 (internal) and 0.86 (external) for AS detection, demonstrating its potential for early disease screening and improved clinical decision-making.

In ref. [43], the authors investigate the effectiveness of a deep learning-based multimodal risk assessment for patients with IHD using ECGs and CXRs. The dataset consists of 2107 patients who underwent elective percutaneous coronary intervention (PCI) at The University of Tokyo Hospital, with raw 12-lead ECG recordings and standardized 256 × 256 CXR images. The study applies DNNs trained to detect LVSD from ECGs and cardiomegaly from CXRs, leveraging previously validated models and training with the VinDr-CXR dataset for cardiomegaly detection. The study follows paradigms of multimodal deep learning, explainability, and risk stratification, integrating ECG and CXR data to classify patients into four risk groups: dual-modality high-risk, ECG high-risk, CXR high-risk, and no-risk. The primary focus is on predicting MACEs such as cardiac death, stroke, acute coronary syndrome, and heart failure hospitalization. The results indicate that the dual-modality high-risk group had the highest MACE incidence, with a hazard ratio (HR) of 2.37 (*p* < 0.001) compared to the no-risk group, demonstrating the potential of deep learning for comprehensive cardiovascular risk assessment.

In ref. [60], the authors in this paper propose a novel approach for heart disease detection using Motif transformation-based Continuous Wavelet Transform (CWT) time-frequency images with DenseNet deep transfer learning. The dataset used in this study comprises 162 ECG recordings from the MIT-BIH and BIDMC databases, representing three categories: arrhythmia (ARR), normal sinus rhythm (NSR), and congestive heart failure (CHF). The proposed method follows a multi-step process, starting with denoising and segmentation of ECG signals, followed by the application of Motif Transformation (MT), which extracts distinctive patterns within the ECG signals. These transformed signals are then subjected to CWT, generating scalogram images that capture both time and frequency domain characteristics. Finally, the scalogram images are classified using DenseNet deep transfer learning models (DenseNet121, DenseNet169, and DenseNet201). The study employs transfer learning as a key paradigm to enhance model performance with limited training data, alongside explainability techniques such as Grad-CAM to visualize critical areas of the ECG signals influencing classification decisions. The primary heart diseases addressed include arrhythmias and congestive heart failure, both of which can lead to severe cardiac complications. The proposed MT + CWT + DenseNet model achieves an impressive accuracy of 99.31%, outperforming traditional CWT + DenseNet models and demonstrating the potential of motif transformation in improving ECG-based heart disease detection.

In Table 7, many ECG-based deep learning studies do not explore advanced paradigms such as federated learning, uncertainty quantification, or explainability beyond basic saliency mapping, while CNNs and DenseNet dominate, and transformers, GANs, and autoencoders remain underutilized. Data variability across populations, devices, and recording conditions poses generalization challenges, while multimodal integration with other imaging modalities requires extensive computational resources and standardized protocols. Further efforts are needed to improve model interpretability, enable real-time monitoring with wearable devices, and ensure reliability through uncertainty quantification in clinical settings.

### 6.3. Discussion of Research Gaps in Electrocardiogram Imaging and Deep Learning

Despite these advancements, several challenges remain in ECG-based deep learning. Data variability and generalization remain significant hurdles, as ECG signals differ across populations, devices, and recording conditions, necessitating large, diverse datasets for robust models. Model interpretability and clinical acceptance also pose challenges, as black box deep learning approaches may lack transparency, making it difficult for clinicians to trust automated predictions. Additionally, multimodal integration of ECGs with other imaging or clinical data requires extensive computational resources and standardized protocols. However, recent developments in transfer learning and explainability techniques, such as Grad-CAM, have helped bridge the gap by improving model performance with limited data and enhancing result interpretability. Future research should focus on federated learning for privacy-preserving model training, real-time ECG monitoring with wearable devices, and uncertainty quantification to ensure reliability in clinical settings.

When comparing these studies collectively, it becomes clear that model performance varies significantly depending on the task and dataset characteristics. For example, ref. [42] reported AUROC values ranging between 0.81 and 0.89 for valvular disease detection, reflecting strong but not perfect generalization, particularly when tested across external cohorts. In contrast, ref. [43] emphasized the prognostic value of multimodal integration, where combining ECG and chest X-ray data improved risk stratification for major adverse cardiovascular events, yielding clinically relevant hazard ratios rather than pure classification accuracy. On the other hand, ref. [60] achieved strikingly high accuracy (99.31%) in arrhythmia and heart failure classification, though this result was obtained on smaller benchmark datasets and may not fully represent performance in real-world heterogeneous populations. Taken together, these findings suggest that while deep learning models can achieve near-perfect results in controlled tasks with limited scope, their generalization across diverse diseases, populations, and clinical settings remains more modest. This reinforces the need for large-scale, multi-institutional studies and standardized evaluation protocols to establish reliable benchmarks and ensure the clinical utility of ECG-based deep learning systems.

ECG-based deep learning models have demonstrated high predictive performance, yet latency and methodological limitations remain critical considerations for real-world deployment. Large-scale studies such as [42], which analyzed over 617,000 ECGs paired with TTEs, achieved AUROC values up to 0.89 but required substantial preprocessing and deep learning pipelines, potentially introducing computational delays that may limit real-time application. Multimodal approaches integrating ECGs with chest X-rays [43] enhanced prognostic accuracy for major adverse cardiovascular events but added complexity and inference time due to the dual-modality processing and risk stratification framework. High-accuracy arrhythmia detection using DenseNet-based transfer learning [43] reached 99.31% on benchmark datasets; however, the small cohort size and offline processing setup limit conclusions regarding latency and deployment feasibility in clinical or wearable scenarios. Additionally, variations in signal quality across devices and populations introduce robustness challenges, as models trained on controlled datasets may experience degraded performance in heterogeneous settings. Collectively, these findings indicate that while ECG deep learning models excel in predictive accuracy, careful optimization for computational efficiency, real-time inference, and cross-population generalization remains essential to ensure practical clinical utility.

## 7. Other Image Modalities

There are rare studies such as [61,62,63,64,65,66,67,68] that range from using phonocardiograms (PCGs), infrared thermal imaging, tongue image analysis, and human-engineered heart tissues to detect heart diseases. These approaches provide alternative diagnostic methods compared to imaging techniques like US, X-ray, MRI, CT, and ECG. The study on Deep Recurrent Learning for Heart Sounds Segmentation introduces a Long Short-Term Memory (LSTM) network with Fourier Synchrosqueezed Transform (FSST) to segment heart sounds in PCGs, demonstrating high sensitivity and accuracy [61]. Similarly, Rheumatic Heart Disease (RHD) detection using deep learning on spectro-temporal heart sounds applies a CNN trained on Mel spectrograms, achieving 96.1% accuracy without requiring prior segmentation [62]. These methods show potential in non-invasive cardiac screening, particularly in regions where echocardiography or ECGs are less accessible.

Another innovative approach is circadian assessment of heart failure, which incorporates Heart Rate Variability (HRV) data and clinical parameters into polar images, enabling a 2D deep learning model to assess heart failure risk with an AUROC of 0.883 [62]. This technique enhances current diagnostic tools by integrating time-based variations, providing a more holistic view of cardiac function. Similarly, tongue image analysis for heart disease detection combines Simulated Annealing with EfficientNet-based deep learning, achieving a 99.3% accuracy in disease classification, demonstrating the potential of traditional medicine-inspired diagnostics in modern AI-driven healthcare [64]. Additionally, a study on deep learning for heart attack detection from digital images employs a modified ResNet-50 to classify individuals experiencing heart attacks based on body posture, achieving 92% accuracy, which could be crucial for emergency medical interventions [64].

Other novel methodologies include automated assessment of Engineered Heart Tissues (EHTs) for drug screening, where a deep learning and template-matching approach is used to track contractile properties of patient-derived heart tissues [66]. This method enhances in vitro testing for cardiotoxicity, offering an early-phase screening tool in drug development. Furthermore, thermal imaging-based remote heart rate and blood pressure estimation bypasses the limitations of contact-based methods like ECG and PPG, using infrared facial imaging and CNN models (DEWNet) to track microtemperature changes due to blood flow, demonstrating feasibility with a mean error of 13.592 bpm [67]. This approach holds promise for non-contact vital sign monitoring, particularly in remote healthcare settings.

Nuclear medicine imaging, including Single-Photon Emission Computed Tomography and Positron Emission Tomography, represents a promising frontier in cardiac diagnostics, offering unique functional insights into myocardial perfusion, viability, and molecular processes through radiopharmaceutical uptake visualization. The integration of artificial intelligence has accelerated advancements across the imaging workflow, from acquisition enhancement to automated interpretation and risk prediction. Deep learning models improve image quality while enabling reduced radiation doses and acquisition times, while machine learning algorithms facilitate automated quantification of perfusion deficits and ventricular function. Large-scale initiatives have validated artificial intelligence models that predict coronary artery disease and major adverse cardiac events with greater accuracy than conventional methods. Beyond perfusion assessment, artificial intelligence shows promise in analyzing novel radiotracers and integrating hybrid imaging data for comprehensive risk profiling. For a detailed and comprehensive examination of artificial intelligence in nuclear cardiac imaging, including specific techniques, model architectures, and recent clinical trials, readers are directed to the excellent review in [69] which provides an in-depth exploration of this rapidly evolving field and its transformative potential in cardiovascular care.

Although these methodologies show great promise, they also present several challenges. One major obstacle is the availability and standardization of data, as many of these approaches rely on small, specialized datasets, which restrict their generalizability and global applicability. Additionally, the interpretability of deep learning models remains a concern, as their black box nature makes it difficult for clinicians to fully trust the predictions. Validating these models in clinical settings and ensuring seamless integration with existing diagnostic tools also require further research and extensive testing before they can be widely adopted. Nevertheless, these alternative techniques open new possibilities for heart disease detection, particularly in non-invasive, real-time, and personalized healthcare. Future advancements should emphasize multimodal data integration and the real-time implementation of these models in wearable and telemedicine technologies.

## 8. Overall Discussion and Future Pathways

The rapid advancements in deep learning have significantly transformed cardiac imaging, enabling more precise and automated heart disease diagnosis. This section provides a comprehensive discussion of the reviewed studies, highlighting key findings, methodological strengths, and existing challenges. Additionally, it outlines future research opportunities to enhance model performance, interpretability, and clinical integration, ensuring that deep learning-driven diagnostics continue to evolve toward more reliable and widely adopted solutions in cardiovascular healthcare.

### 8.1. Global Discussion of Reviewing Results

This section provides a global discussion of the reviewed studies, synthesizing the key insights gained from deep learning applications in cardiac imaging. It highlights the major achievements, such as improvements in accuracy, segmentation, and real-time diagnostic capabilities, while also addressing the limitations and challenges that remain. In addition, the discussion considers the computational demands of different approaches, emerging modalities, and strategies for dealing with rare conditions and limited datasets. Broader themes such as clinical translation, resource efficiency, and the potential of multimodal fusion are also emphasized, offering a comprehensive perspective on both the current state of research and future directions in this rapidly evolving field.

#### 8.1.1. Distinguishing Experimental Results from Clinical Validation

A critical distinction emerging from this review is the gap between experimental performance and clinical validation. While numerous studies report high accuracy on benchmark datasets such as the CAMUS dataset, these results are predominantly retrospective and derived from curated data. Clinically validated models remain comparatively scarce. Notable exceptions include deep learning models from the registry of fast myocardial perfusion imaging with next-generation single-photon emission computed tomography, which have undergone multi-center prospective validation for predicting major adverse cardiac events. Similarly, the EchoNet-Dynamic model demonstrated diagnostic performance for heart failure that was comparable to or exceeding experienced cardiologists in prospective assessment. Prospective studies further exemplify the pathway from algorithmic development to clinical evaluation. For most modalities and applications, however, prospective trials remain limited, and generalizability across diverse populations, imaging protocols, and equipment vendors requires urgent investigation.

#### 8.1.2. Progress in Deep Learning for Cardiac Imaging

This review of deep learning applications in heart disease detection across various imaging modalities highlights significant progress in accuracy, segmentation, and classification. Studies leveraging ultrasound, X-ray, MRI, CT, ECG, and alternative methods such as phonocardiograms and thermal imaging demonstrate the adaptability of deep learning in diverse clinical contexts. CNN-based architectures, including UNet, ResNet-50, and VGG16, are widely applied, with hybrid models and multi-task learning further enhancing segmentation performance. Transfer learning and interpretability techniques, such as Grad-CAM and saliency mapping, have improved model transparency, while multimodal learning has enabled more comprehensive risk stratification. Reported accuracy metrics, such as AUROC values ranging from 0.81 to 0.95 and DSC exceeding 0.9, emphasize the potential of deep learning to advance cardiac diagnostics. These methodologies have shown promise in detecting a wide range of cardiac conditions, from congenital defects to heart failure and valvular diseases. Additionally, recent advancements in real-time deep learning models have demonstrated superior diagnostic performance compared to traditional manual methods in clinical settings. For example, deep learning models reviewed in Section 2.2 and Section 3.2, such as the EchoNet-Dynamic model [38], or even [28] achieved AUROC values of about 0.95 to 1.0 (approaching 1.0) for heart failure diagnosis, surpassing the performance of experienced cardiologists in some cases. In contrast, traditional diagnostic workflows often rely on manual interpretation, which is susceptible to observer variability and slower turnaround. Furthermore, real-time deep learning models enable instantaneous image analysis, significantly reducing diagnostic latency and enabling decision support in time-sensitive clinical scenarios. These attributes illustrate the potential of deep learning models not only to match but also to improve upon existing diagnostic benchmarks. It is important to note that the accuracy metrics reported across studies (e.g., AUROC, DSC) are derived from heterogeneous datasets, imaging protocols, and experimental conditions, and therefore should not be interpreted as directly comparable. They are presented here to illustrate the range of achievable performance rather than to establish superiority between methods.

#### 8.1.3. Computational Efficiency and Resource Considerations

While the specific model size (e.g., number of trainable parameters or exact architectural depth) is not explicitly reported in most of the reviewed papers, we note that nearly all studies utilize transfer learning and rely on widely adopted architectures such as VGG16, ResNet50, UNet, or EfficientNet. These models typically range from approximately 14 million to 60 million parameters, depending on customization and task complexity. This range suggests a moderate computational footprint, commonly feasible on standard CPU systems or low-end GPUs. Moreover, many of these models were implemented using platforms such as TensorFlow, PyTorch, or Keras, which support efficient deployment and real-time inference, especially when optimized with techniques like quantization or pruning. Therefore, although exact model sizes are not always provided, the choice of architectures and platforms indicates that most reviewed methods are generally real-time capable or near real-time in typical clinical settings.

#### 8.1.4. Research Gaps and Methodological Challenges

Despite these advancements, several research gaps and challenges remain, especially when compared to advancements in brain tumor detection, for instance [91]. Many studies rely on relatively small or specialized datasets, limiting the generalizability of trained models. While CNNs dominate, underutilized techniques such as transformers, GANs, and autoencoders could offer improved feature extraction and robustness. Additionally, uncertainty quantification and explainability remain critical concerns, as black box models hinder clinical trust and adoption. The integration of federated learning could address data privacy concerns in medical artificial intelligence, while reinforcement learning may optimize decision-making processes in automated diagnostics. Furthermore, hyperparameter tuning in many studies remains empirical rather than systematic, leaving room for optimization through Bayesian learning or adaptive algorithms.

#### 8.1.5. Emerging Modalities and Novel Applications

Emerging methodologies using alternative imaging techniques present new opportunities but require further validation and standardization. Phonocardiograms and tongue image analysis have shown high classification accuracy, yet their clinical application remains limited. Infrared thermal imaging for heart rate and blood pressure monitoring offers a promising non-contact alternative to ECG and PPG, but its feasibility for large-scale deployment needs to be explored further. The application of deep learning in Engineered Heart Tissues (EHTs) has demonstrated its potential for drug screening and cardiotoxicity assessment, yet challenges in standardization and scalability persist. To fully integrate these novel techniques into routine clinical practice, future research should focus on multimodal data fusion, real-time model deployment in wearable devices, and advanced interpretability frameworks to ensure reliability and trustworthiness in artificial intelligence-driven cardiac diagnostics.

#### 8.1.6. Handling Rare Cardiac Conditions and Data Augmentation

Moreover, handling rare cardiac conditions presents a significant challenge in deep learning due to the limited availability of labeled data. Unlike common heart diseases, rare conditions often lack sufficient training samples, which can hinder model generalization. To address this, researchers have adopted strategies such as transfer learning (e.g., [44]), where models pre-trained on large, related datasets are fine-tuned on smaller, task-specific datasets. Data augmentation techniques are also employed to synthetically expand datasets through transformations such as rotation (e.g., [44]), scaling, or flipping. Additionally, generative models (e.g., [46]) like GANs can create realistic synthetic images to supplement scarce data. Emerging approaches like few-shot learning and meta-learning further aim to train models to recognize new classes from minimal examples. Despite these advancements, improving performance for rare conditions remains a priority, necessitating collaboration on data sharing and the development of inclusive, multi-institutional datasets. As discussed, data augmentation plays a critical role in enhancing the performance of deep learning models in cardiac imaging, especially when dealing with limited datasets. By applying transformations such as rotation, scaling, flipping, contrast adjustment, and elastic deformation, data augmentation increases the diversity of training samples without requiring additional labeled data. This helps prevent overfitting, improves model generalization, and is particularly effective in conditions with class imbalance or rare cardiac abnormalities. For example, augmentation has been shown to significantly improve segmentation accuracy in ultrasound and X-ray imaging tasks (see Section 2.2 and Section 3.2). However, augmentation must be applied carefully because unrealistic or excessive transformations can introduce artifacts or distort clinically relevant features, potentially reducing diagnostic accuracy. As such, domain-specific augmentation strategies, often combined with automated hyperparameter tuning, are essential for achieving optimal results.

#### 8.1.7. Multimodal Fusion for Improved Diagnostics

Additionally, multimodal fusion in cardiac diagnostics, particularly the integration of ECG and echocardiographic data, has emerged as a powerful approach to improve diagnostic accuracy. Each modality provides unique physiological insights: ECG captures electrical activity, while echocardiography offers detailed structural and functional imaging. When combined using deep learning models, these complementary data sources enable richer feature representations and improved classification performance. For example, the studies reviewed in [42] demonstrate that fusion models outperform single-modality models in predicting valvular dysfunction and major adverse cardiac events, with AUROC values improving by up to 10–15%. Deep learning architectures such as multi-stream CNNs, attention-based fusion layers, and shared encoder–decoder networks have been increasingly adopted for this purpose. Despite their promise, multimodal systems require careful alignment and synchronization of heterogeneous data, and managing missing or noisy inputs remains a technical challenge. Nonetheless, multimodal fusion holds strong potential to enhance reliability and robustness in AI-driven cardiac diagnostics.

#### 8.1.8. Clinical Translation and Future Opportunities

Despite the strong progress demonstrated, several challenges remain before these models can be routinely implemented in clinical practice. Many studies still rely on controlled experimental datasets that may not capture real-world variability, and the lack of large-scale, standardized multi-center datasets limits generalizability. Integration with existing clinical workflows also remains underdeveloped. Looking forward, opportunities lie in creating interoperable artificial intelligence platforms that connect seamlessly with hospital systems, establishing continuous validation pipelines through real-world feedback, and fostering stronger collaboration between clinicians, engineers, and policymakers to ensure that technical advances align with clinical needs. Prospective trials provide a model for how multi-center collaboration can accelerate clinically meaningful validation.

#### 8.1.9. Resource-Constrained Platforms

Recent advances in deep learning for cardiac imaging have increasingly emphasized the feasibility of deploying models on resource-constrained platforms such as portable ultrasound systems, embedded devices, and low-power GPUs. In ultrasound imaging, models such as CNN-based echocardiography pipelines [37] have been optimized through efficient spatio-temporal processing, pruning, and lightweight segmentation networks to enable real-time analysis of echocardiographic data without reliance on high-end computational resources. Similarly, hybrid echocardiography–MRI approaches [28], have demonstrated that streamlined architectures with regression and classification branches can achieve competitive performance on clinical tasks while remaining suitable for deployment on limited hardware. In coronary artery analysis, 3D UNet and DBN frameworks [29] have shown that careful design of segmentation and regression modules can reduce computational overhead, enabling implementation on GPUs with modest memory capacity.

In computed tomography, segmentation and classification models such as 3D UNet–based pipelines [51] and optimized frameworks employing CLAHE preprocessing, SAT-Net, and EESNN classifiers [53] have incorporated strategies like patch-based training, downsampling, and residual block optimization to minimize memory usage while maintaining high segmentation accuracy. Furthermore, multimodal CT–MRI integration models [54] leveraging orthogonal UNets with statistical shape modeling have reduced inference time and improved efficiency, demonstrating the potential of quantization and shape-context refinement to lower resource demands without sacrificing diagnostic accuracy. Collectively, these studies highlight that across ultrasound, CT, and multimodal imaging, deep learning methods are not only advancing in predictive power but also evolving toward computationally efficient designs, making them practical for adoption in real-world healthcare environments where resources are often constrained.

### 8.2. Future Opportunities

The field of deep learning for heart disease detection continues to evolve, presenting numerous opportunities to enhance model performance, robustness, and clinical applicability. Future research should focus on improving the adaptability of deep learning models to diverse patient populations, optimizing computational efficiency for real-time applications, and integrating multiple data sources to create more comprehensive diagnostic tools. Additionally, addressing the existing challenges in interpretability, privacy, and data scarcity will be crucial in ensuring reliable and trustworthy artificial intelligence-driven healthcare solutions.

#### 8.2.1. Strengthening Clinical Validation Through Prospective Studies

The most pressing need is rigorous prospective validation of deep learning models in diverse, real-world clinical settings. Multi-center registries provide a valuable template for developing and externally validating models. Future prospective studies should evaluate not only diagnostic accuracy but also clinical workflow integration, impact on decision-making, and patient outcomes. Initiatives that combine imaging data with electronic health records and long-term follow-up will be essential for demonstrating clinical utility.

#### 8.2.2. Exploring Advanced Deep Learning Models

Recent advancements in deep learning, such as transformers, attention mechanisms, and domain adaptation, have demonstrated significant promise in medical image analysis, yet their full potential in cardiac imaging remains underexplored. Transformers and Vision Transformers, for example, have shown superior performance in capturing long-range dependencies and context awareness in images, yet their application to cardiac imaging tasks requires further exploration. Attention mechanisms hold great potential to enhance feature extraction by prioritizing clinically relevant regions, which could substantially improve classification and segmentation accuracy, but their widespread adoption in cardiac diagnostics is still limited. Additionally, Graph Neural Networks (GNNs) offer a powerful framework for modeling complex cardiac structures and spatial relationships in imaging data, but this promising approach has not been fully utilized in heart disease detection. The use of various autoencoders, such as variational, denoising, and sparse autoencoders, also presents an exciting opportunity to improve feature learning and robustness, especially in situations where labeled data are scarce. In particular, sparse deep networks are emerging as an effective strategy for reducing computational complexity, yet their potential for real-time cardiac imaging applications remains largely untapped. Future research must focus on harnessing the power of these techniques to unlock new levels of accuracy, efficiency, and adaptability in heart disease diagnosis, as these areas are currently underexplored and offer substantial room for improvement.

A crucial challenge in deploying deep learning models for cardiac imaging lies in balancing three competing objectives: accuracy, interpretability, and computational efficiency. State-of-the-art models such as CNNs and transformers often achieve high accuracy. For example, AUROC values exceed 0.95, as demonstrated in [28], for instance, but they require substantial computational resources and are often viewed as “black boxes” in clinical environments. This lack of interpretability limits clinician trust and complicates regulatory approval. On the other hand, simpler models or rule-based systems offer high interpretability and real-time efficiency but tend to underperform on complex tasks such as multi-class classification or fine-grained segmentation. Trade-offs also emerge when choosing between real-time deployment (e.g., on portable devices) and model complexity. Recent approaches like attention mechanisms, explainability techniques (e.g., Grad-CAM, saliency maps), and lightweight models (e.g., MobileNet, EfficientNet) aim to mitigate these conflicts by improving transparency and reducing computational overhead while retaining reasonable accuracy. Future research should continue to explore this balance, especially in high-stakes domains like cardiology.

Transformers, graph neural networks, and autoencoders present promising directions for cardiac imaging, but their adoption is still limited. Current challenges include the high computational demands of these models, their increased complexity that reduces interpretability, and the lack of adaptation of large-scale pre-training strategies to cardiac data. Future opportunities involve developing hybrid or sparse architectures that balance accuracy with efficiency, adapting foundation models from general vision tasks to cardiovascular imaging, and advancing compression or distillation techniques that enable deployment in resource-constrained and real-time environments.

#### 8.2.3. Use of Automatic Tuning and Hyperparameter Optimization

Hyperparameter selection remains one of the most time-consuming aspects of deep learning model development. Instead of relying on manual tuning, automated approaches such as Bayesian optimization, genetic algorithms, and reinforcement learning-based hyperparameter search can significantly enhance model efficiency. These methods can dynamically adapt learning rates, batch sizes, and network architectures, optimizing performance while reducing computational cost. Automated hyperparameter tuning also allows models to generalize better across different datasets and patient populations, making them more applicable in real-world clinical settings.

Automated hyperparameter optimization can enhance performance, but it is often computationally expensive and prone to overfitting when applied to small or imbalanced datasets. Moreover, many frameworks are not designed with the unique characteristics of medical data in mind. Future research should focus on developing domain-aware optimization approaches that incorporate clinical knowledge, designing adaptive strategies for heterogeneous datasets, and embedding these methods within federated learning environments to enable scalable and privacy-preserving model refinement across institutions.

#### 8.2.4. Real-Time Uncertainty Quantification

Deep learning models in medical imaging should not only provide predictions but also quantify uncertainty to improve clinical trust and decision-making. Bayesian deep learning techniques, such as Monte Carlo dropout and variational inference, can be integrated to assess prediction confidence and highlight uncertain cases that require expert review. Uncertainty-aware models can help reduce false positives and negatives by flagging ambiguous cases, ensuring that diagnostics are both reliable and clinically interpretable. Future research should focus on real-time uncertainty estimation, allowing deep learning models to assess confidence levels dynamically during image analysis.

Uncertainty-aware models are essential for clinical decision-making, but existing methods often increase inference time and lack standardized benchmarks for evaluation. Their integration into clinical workflows also remains limited. Future opportunities lie in developing lightweight uncertainty estimation techniques suitable for real-time use, establishing calibration frameworks that ensure confidence scores are clinically meaningful, and embedding uncertainty-aware outputs into electronic health records to support patient-specific risk assessment and decision-making.

#### 8.2.5. Real-Time Interpretability and Explainability

For deep learning-based heart disease detection to gain widespread clinical adoption, it is essential to enhance model interpretability. Existing techniques like Grad-CAM, SHAP values, and saliency maps can visualize which image regions influence predictions, helping clinicians understand the model’s decision-making process. However, real-time interpretability methods must be improved to offer interactive and adaptive explanations, ensuring that models can provide justifications for their classifications in a clinically meaningful way. Future work should explore hybrid interpretability frameworks that combine global explanations (overall model behavior) with local interpretability (individual predictions) to create more transparent and trustworthy diagnostic tools.

In addition, the application of artificial intelligence in cardiac diagnostics introduces several ethical and practical risks that must be addressed before widespread clinical adoption. One major concern is algorithmic bias, where models trained on non-representative datasets may underperform on minority populations, potentially exacerbating healthcare disparities. Lack of transparency in decision-making (especially with deep learning models often perceived as black boxes) can undermine clinician trust and impede regulatory approval. Additionally, patient data privacy is a critical issue, particularly with the large-scale data aggregation required to train robust models. Informed consent for AI-driven decisions and clinical accountability in case of misdiagnosis remain legally and ethically complex. As a result, integrating artificial intelligence into healthcare systems requires rigorous validation, clear regulatory frameworks, and the development of explainable and fair models to ensure safety, equity, and trust.

Interpretability remains a bottleneck for clinical adoption, as many existing methods provide post hoc explanations that may not fully capture the reasoning process of the model. Visual explanation maps are often ambiguous and require additional clinical validation, and most approaches lack the interactivity needed in real-time diagnostic settings. Promising opportunities include co-developing interpretability tools with clinicians to align them with diagnostic reasoning, advancing hybrid frameworks that combine global and local explanations for improved transparency, and creating interactive systems where clinicians can query and refine model predictions dynamically.

#### 8.2.6. Federated Learning and Privacy-Preserving

With increasing concerns over data privacy in medical imaging, federated learning presents a promising solution for collaborative model training without sharing sensitive patient data. By enabling hospitals and institutions to train models on decentralized datasets while keeping data local, federated learning can enhance generalizability while preserving patient confidentiality. Differential privacy techniques and homomorphic encryption can further secure patient data during model training, ensuring compliance with regulations such as GDPR and HIPAA. Future advancements should focus on optimizing communication efficiency in federated learning frameworks, reducing computational overhead, and improving model aggregation strategies to enhance performance across diverse healthcare settings.

Federated learning offers a pathway for secure model training, but its effectiveness is often limited by heterogeneous data across institutions, communication overhead, and inconsistent labeling protocols. These issues can hinder both performance and scalability. Future opportunities include the development of adaptive aggregation methods that account for institutional variability, the integration of blockchain for secure traceability of model updates, and the combination of federated learning with self-supervised pre-training to improve generalization while reducing reliance on large amounts of labeled data.

#### 8.2.7. Potential Impact of Image Compression Techniques

Efficient image compression is crucial for reducing storage requirements and improving processing speed in deep learning-based medical imaging. Techniques such as compressed sensing, sparse coding, and wavelet transforms can retain essential image features while minimizing redundancy. These approaches can be particularly beneficial for real-time applications, enabling faster model inference on low-power devices like portable ultrasound machines and wearable ECG monitors. However, excessive compression can lead to loss of critical diagnostic information, affecting model accuracy. Future research should focus on balancing compression efficiency with diagnostic integrity, ensuring that deep learning models retain high performance even with reduced data sizes.

Image compression can accelerate deep learning workflows, but excessive compression risks eliminating subtle diagnostic features, and current evaluation metrics such as PSNR or SSIM do not fully reflect clinical relevance. There is also a lack of task-specific compression frameworks optimized for diagnostic performance. Future opportunities include the design of clinically informed compression methods that preserve disease-relevant features, the integration of compression with edge computing to enable real-time analysis on portable and wearable devices, and the refinement of compression schemes that strike an optimal balance between efficiency and diagnostic integrity.

## 9. Conclusions

This review highlights the substantial advancements in deep learning applications for heart disease detection across a wide range of imaging modalities, including ultrasound, X-ray, MRI, CT, ECG, and emerging approaches such as phonocardiograms, thermal imaging, and tongue image analysis. These studies demonstrate the potential of deep learning to improve diagnostic accuracy, segmentation quality, and risk stratification, with models achieving high AUROC scores and Dice coefficients in various cardiac tasks. The integration of CNNs, attention mechanisms, and transfer learning has further enhanced performance, while multimodal fusion approaches have enabled more comprehensive evaluations of cardiovascular conditions. At the same time, several persistent challenges must be addressed before widespread clinical adoption can be achieved. Many studies still rely on small or highly curated datasets, limiting generalizability to diverse patient populations. Interpretability and uncertainty quantification remain underdeveloped, and data scarcity for rare cardiac conditions continues to hinder progress. In addition, privacy concerns, interoperability with existing clinical systems, and the regulatory complexity of AI-driven diagnostics present non-trivial barriers. Looking ahead, future opportunities are abundant. Advances in transformers, graph neural networks, and autoencoders could unlock new levels of accuracy and efficiency, while federated learning and privacy-preserving techniques provide promising solutions for secure large-scale collaboration. The development of domain-aware hyperparameter optimization, real-time uncertainty estimation, and interactive interpretability frameworks will be crucial in bridging the gap between model performance and clinician trust. Furthermore, integrating task-specific image compression with edge computing and wearable devices could enable scalable, real-time cardiac monitoring beyond the clinic. By addressing these gaps and leveraging emerging techniques, deep learning has the potential to revolutionize cardiac diagnostics. Achieving this vision will require not only technical innovation but also collaborative efforts between researchers, clinicians, and policymakers to ensure that AI-driven solutions are accurate, transparent, secure, and equitable. With continued progress, deep learning can contribute to earlier detection, more reliable monitoring, and ultimately improved outcomes in cardiovascular health.

## Figures and Tables

**Table 1:** Overview of the key aspects of review studies on deep learning and cardiac diseases.

Reference	Context	Revealed Advantages	Limitations
[26]	Review of deep learning applications in cardiovascular image analysis (MRI, CT, US) with focus on CNNs and GANs.	High performance comparable to experienced physicians, potential for clinical integration.	Outdated techniques; overlooks recent advancements (transformers, attention mechanisms, transfer learning); neglects data quality, compression, and generalizability issues.
[25]	Review of deep learning techniques for cardiac image segmentation (MRI, CT, US), focusing on CNNs, FCNs, UNet.	Precision and reproducibility through public datasets.	Lacks discussion on classification, feature extraction, recent advancements, data preprocessing, augmentation, and multimodal integration.
[24]	Review of AI in diagnosing heart failure (echocardiography, MRI, ECG), focusing on decision trees, CNNs, RNNs.	Improved diagnostic accuracy and early detection.	Limited focus on image-specific tasks, lacks deep analysis of advanced deep learning techniques.
[11]	Global overview of multimodality cardiac image computing, briefly mentioning deep learning.	Broad perspective on multimodality image computing.	Lacks detailed exploration of deep learning techniques in cardiac image analysis.

**Table 2:** Categorization of reviewed papers by imaging modality and number of references.

Ref.	Generalized Category	Num. of References
[28,29,30,31,32,33,34,35,36,37,38,39,40,41,42]	US	15
[43,44,45,46,47,48,49,50]	X-ray	8
[51,52,53,54,55]	MRI	5
[56,57,58,59]	CT	4
[42,43,60]	ECG	3
[61,62,63,64,65,66,67,68]	Digital photos, microscopy, thermal images, audio and others	8

**Table 3:** Summary of research on ultrasound imaging and deep learning for heart disease detection.

Reference	Year	Dataset	Methods	Paradigms	Diseases	Accuracy
[28]	2019	25,776 patients (1026 mortalities). 20,651 (Hospital A), 1560 (Hospital B).	Text mining, 3 layers deep learning, TensorFlow.	Noted black box nature of use deep learning model.	CHD, HF (ICD-10 codes).	AUROC: 0.912 internal, 0.898 external, 0.958 CHD, 0.913 HF).
[29]	2021	1149 fetal heart images from ultrasound videos (18–24 weeks gestation). Includes four standard views (4CH, 3VT, LVOT, RVOT) and three CHDs (ASD, VSD, AVSD).	Mask R-CNN, ResNet50 backbone. Labeling with LabelMe.	Transfer learning: Pre-trained on Microsoft COCO dataset. Multi-task learning.	CHDs: ASD, VSD, and AVSD.	MAP: 98.30% (intra-patient), 82.42% (inter-patient). IOU: 79.97%. DSC: 89.70%.
[30]	2024	Retrospective cohort from Taiwan (2011–2022)	Four-step deep learning approach: (1) VGG16 for image quality assessment and view classification, (2) UNet for IVS auto-segmentation, (3) LSTM for dynamic radiomics analysis.	Interpretability through radiomics, segmentation techniques.	FC, HCM, CA.	Image quality assessment: 91.7%, View classification: 95.2%, Auto-segmentation: Dice score 0.88, LSTM classification: 74.5%.
[31]	2024	50 patients’ echocardiographic recordings at HR 50–140 beats/min.	Automated LVGLS using DL. Linear mixed model for trend analysis.	/	SND.	Strong heart rate LVGLS correlation (*p* < 0.001). 32% LVGLS reduction with heart rates increase.
[32]	2017	91 fetal heart US videos (12 subjects, 20–35 weeks). 4C, LVOT, 3V views.	SBM (AlexNet, VGGNet), DTE, HTE for spatio-temporal aggregation.	Multi-task learning for visibility, view, and localization.	CHD detection.	Classification: 88.27% (LVOT), 79.80% (3V) with HTE. Localization: 20.32% error (HTE, IOU > 0.25).
[33]	2024	2556 echocardiography and 1453 CMRs include CH, 2CH, and PLAX views.	Video-based CNN, binary cross-entropy loss for classification, mean squared error for regression AdamW optimizer with early stopping.	/	Conditions associated with myocardial fibrosis, inflammation, and infiltration.	WMA: AUROC 0.873. LGE: AUROC 0.699. T1: AUROC 0.614. T2: AUROC 0.553. ECV: AUROC 0.564.
[34]	2020	CTA images (34 subjects, no centerline) and Rotterdam Coronary Artery Algorithm Evaluation (18 subjects, with centerline)	3D UNet for coronary artery segmentation + DBN for feature extraction and regression	Not explicitly mentioned	Coronary artery disease (CAD), plaque, stenosis	DSC: 0.8291
[35]	2021	259 elderly sarcopenia patients.	CNN for image optimization. ReLu/swish activation.	/	CHF with sarcopenia correlation.	PSNR = 20, SSIM = 0.09. Diagnosis similarity: 93.5% (experimental) vs. 87.0% (control).
[36]	2021	161 TTE 3DE images from 129 HLHS patients.	FCN (V-Net), input configs (single-phase, two-phase, four-phase, CSP), preprocessing (resampling, orientation)	/	HLHS, Tricuspid Valve Segmentation	Median DSC: 0.86, MBD: 0.35 mm (merged); Avg DSC: 0.77, MBD: 0.6 mm (individual); Commissural landmarks improved MBD to 0.38 mm.
[37]	2021	80 elderly patients with ALHF (August 2017–February 2019)	CNN for image preprocessing (binarized threshold segmentation, illumination processing), feature extraction, and classification.	/	ALHF	Diagnostic coincidence rate: 93.94% (observation group) vs. 74.29% (control group); higher specificity, sensitivity, and accuracy (*p* < 0.05)
[38]	2023	EchoNet-Dynamic, CAMUS, and local dataset.	Deep spatio-temporal model: regression + classification, dynamic/static data processing	/	Heart Failure (HFrEF, EF < 40%)	AUROC: 0.95 (dynamic), 1 (validation); static accuracies: 85%, 81%, 93%, 92% (1, 2, 4, 8 images)
[39]	2023	CAMUS dataset (450 patients, 1800 images).	YOLOv7 for chamber detection + UNet for segmentation.	Transfer learning.	Heart failure, myocardial ischemia	DSC: 92.63% (LVENDO), 85.59% (LVEPI), 87.57% (LA); JAC and HD also reported.
[40]	2024	511 fetal heart 3VV images (413 normal, 98 abnormal).	Two-stage: Yolov5 (ROI) + Deeplabv3 (AMFF) for segmentation.	/	CHD: vessel diameter, chamber, arterial, outflow tract, Tetralogy of Fallot.	DSC: 85.55% (PA), 89.12% (Ao), 77.54% (SVC); HD: 3.25; IOU: 74.51%
[41]	2020	153 2D ultrasound images (cardiomyopathy).	UNet CNN for LV segmentation.	/	Cardiomyopathy	DSC: 83.49% (128 × 128), 83.40% (1016 × 708); HD; JC
[42]	2023	617,338 ECGs paired with TTE reports from 123,096 patients (demographically diverse)	NLP pipeline for label extraction; DL model (MLP + EfficientNet CNN) for ECG classification.	Group-stratified k-fold cross-validation; Saliency mapping for interpretability	Aortic Stenosis (AS), Mitral Regurgitation (MR)	AUROC: 0.89 (AS, internal), 0.86 (AS, external); 0.88 (MR, internal), 0.81 (MR, external)

**Table 4:** Summary of research on X-ray imaging and deep learning for heart disease detection.

Reference	Year	Dataset	Methods	Paradigms	Diseases	Accuracy
[43]	2024	2107 patients (Tokyo Univ. Hospital)	DNNs for ECG (LVSD) and CXR (cardiomegaly)	Multimodal deep learning	IHD, LVSD, cardiomegaly	AUROC = 0.77 for PAWP prediction
[44]	2020	NIH ChestX-ray8 (952 images)	CNN (VGG16) with transfer learning and data augmentation	Transfer learning, interpretability (Grad-CAM)	Heart failure (cardiomegaly, congestion)	82% (Sens: 75%, Spec: 94.4%)
[45]	2023	192 heart failure patients (single-center)	ResNet-50 for PAWP prediction, Grad-CAM for explainability	Explainability (Grad-CAM)	HFrEF, HFpEF	82% (Sens: 75%, Spec: 94.4%)
[46]	2023	JSRT and Shenzhen Hospital (862 images)	MWG-UNet (WGAN + UNet with SE blocks)	Generative adversarial learning, multi-task learning	Heart and lung segmentation	DSC: 95.28% (lung), 71.16% (heart); IoU: 74.56% (heart)
[47]	2024	353 heart failure patients (Nanjing Medical Univ.)	CNN, MLP-integrated model	Explainability (Grad-CAM)	Heart failure, cardiomegaly	AUROC: 0.818 (5-year), 0.702 (3-year), 0.706 (1-year)
[48]	2019	NIH Chest X-ray images	CNN (VGG16) with data augmentation and normalization	Transfer learning	Cardiomegaly, emphysema, effusion, hernia, infiltration, mass, nodule, atelectasis, pneumothorax, pleural thickening, pneumonia, fibrosis, edema, consolidation	92.6% (Sens: 92.9%)
[49]	2021	NIH ChestX-ray8 (112,120 images)	CNNs (Xception, DenseNet, MobileNetV2, ResNet-50, VGG16, etc.)	Transfer learning	Heart failure (cardiomegaly)	Xception: 86% (Prec: 77.5%, Rec: 82%, F1: 79%)
[50]	2024	JSRT (247 X-rays) and private dataset (22 images)	ASM + CNN + ADMM-based optimization	Hybrid (model-based deep learning)	Cardiomegaly, pleural effusion, pulmonary edema	DSC: 0.877 ± 0.05, Hausdorff: 17.35 mm ± 4.52 mm

**Table 5:** Summary of research on MRI and deep learning for heart disease detection.

Reference	Year	Dataset	Methods	Paradigms	Diseases	Accuracy
[51]	2016	MICCAI 2012 RV Segmentation Challenge and MICCAI 2009 LV database.	CNN, Autoencoder, and deformable model refinement.	/	Focus on RV/LV segmentation for cardiac function evaluation.	RV: DSC = 0.81 LV: EDV, ESV, EF correlation = 0.99
[52]	2017	MICCAI 2009 database.	Three-step approach: 1. ROI detection using DBNs. 2. Initial segmentation using Otsu’s thresholding. 3. Refinement using DRLS.	/	Focus on LV segmentation for cardiac function evaluation.	Endocardium segmentation: DSC = 0.91. Epicardium segmentation: DSC = 0.94. APD reported for both.
[53]	2022	ACDC, MSD02 and MyoPS 2020 datasets.	nn-TransUNet.	/	LV, RV, MYO, myocardial edema, and scars.	DSC: 93.6% HD: 9.1 mm JSC: 88.3%
[54]	2024	44 patients (cerebral disorders), 335 patients (collected by 2023). cTCD and non-enhanced CMRI used. 3.0T MR scanner (OBL FIESTA CINE 4CH). AW station 4.7 for processing, Pseudo-color coding for PFO and complications.	Deep learning for CMRI analysis.	/	PFO with IAS aneurysm and septum thickening.	Sensitivity: 94.9% Specificity: 60.0% Accuracy: 90.9% PPV: 94.9% NPV: 60.0% AUROC: 0.774
[55]	2024	UK Biobank: 500,000+ participants. 4723 CVD and 5733 healthy controls.	Circle cvi42: Deep learning for CMR analysis. 3D UNet (LC-UNet): Predicts 3D heart chamber volumes from 2D slices.	/	HF, MI-IHD, VAC, CD, AF.	AUROC: 0.918 for LV.

**Table 6:** Summary of related works on computed tomography and deep learning for heart disease detection.

Reference	Year	Dataset	Methods	Paradigms	Diseases	Accuracy
[56]	2016	Multi-center data (*n* = 858) for training; 5677 breast cancer patients for validation	3D UNet deep learning model trained on ECG-gated and low-dose chest CTs	Domain transfer (from cardiovascular radiology to radiation oncology)	Radiation-induced cardiac complications (e.g., coronary artery disease, heart failure)	DSC: 0.95 (training), 0.92 (expert-assisted), 0.89 (real-world clinical use)
[57]	2017	4D contrast-enhanced cardiac CT scans from 1509 patients (21,605 3D images)	3D CNN with residual blocks and batch normalization for whole-heart segmentation	Deep learning, explainability, generalization across cardiac phases	Aortic stenosis, structural heart diseases related to TAVI	DSC: 0.89 ± 0.10, ASSD: 1.43 ± 1.45 mm
[58]	2022	400 CCTA images from two hospitals in Peshawar, Pakistan	CLAHE for contrast enhancement, Modified UNet for segmentation, SAT-Net for feature extraction, RFO for feature selection, EESNN for classification	Deep learning, explainability, optimization	Blood flow-reduced vessels, narrow vessels, blocked vessels, normal vessels	High segmentation and classification accuracy (DSC t and IoU reported)
[59]	2024	MMWHS dataset (20 contrast-enhanced CT scans, 20 contrast-enhanced MRI scans)	Orthogonal 2D UNets for scout segmentation, shape-context estimation with statistical shape models, shape-context-guided UNet for refinement	Deep learning, statistical shape modeling	Left and right ventricles, left and right atria, left ventricular myocardium, ascending aorta, pulmonary artery	DSC: up to 0.935 (CT, left ventricle), 0.895 (MRI, left ventricle).

**Table 7:** Summary of related works on ECGS and deep learning for heart disease detection.

Reference	Year	Dataset	Methods	Paradigms	Diseases	Accuracy
[42]	2023	617,338 ECGs paired with TTEs from 123,096 patients across five hospitals (Mount Sinai Health System)	NLP for label extraction, deep learning model combining MLP and EfficientNet-based CNN	Deep learning, interpretability, external validation	MR, AS	AUROC: 0.88 (internal)/0.81 (external) for MR, 0.89 (internal)/0.86 (external) for AS
[43]	2024	2107 patients who underwent PCI at The University of Tokyo Hospital, with 12-lead ECGs and standardized 256 × 256 CXRs; VinDr-CXR dataset for cardiomegaly detection	DNNs trained to detect LVSD from ECGs and cardiomegaly from CXRs	Multimodal deep learning, explainability, risk stratification	MACEs: cardiac death, stroke, acute coronary syndrome, heart failure hospitalization	Hazard ratio (HR) of 2.37 (*p* < 0.001) for high-risk group compared to no-risk group
[60]	2024	162 ECG recordings from MIT-BIH and BIDMC databases	MT + CWT + DenseNet deep transfer learning (DenseNet121, DenseNet169, DenseNet201)	Transfer learning, Grad-CAM	ARR, NSR, CHF	Accuracy: 99.31%

## Data Availability

The dataset collected and the codes used for data processing, cleaning, and visualization, are publicly available at https://doi.org/10.5281/zenodo.17250312.
